# Consecutive BNT162b2 mRNA vaccination induces short-term epigenetic memory in innate immune cells

**DOI:** 10.1172/jci.insight.163347

**Published:** 2022-11-22

**Authors:** Yuta Yamaguchi, Yasuhiro Kato, Ryuya Edahiro, Jonas N. Søndergaard, Teruaki Murakami, Saori Amiya, Shinichiro Nameki, Yuko Yoshimine, Takayoshi Morita, Yusuke Takeshima, Shuhei Sakakibara, Yoko Naito, Daisuke Motooka, Yu-Chen Liu, Yuya Shirai, Yasutaka Okita, Jun Fujimoto, Haruhiko Hirata, Yoshito Takeda, James B. Wing, Daisuke Okuzaki, Yukinori Okada, Atsushi Kumanogoh

**Affiliations:** 1 Immunopathology, WPI Immunology Frontier Research Center, Osaka University, Suita, Japan.; 2 Department of Respiratory Medicine and Clinical Immunology and; 3 Department of Statistical Genetics, Osaka University Graduate School of Medicine, Suita, Japan.; 4 Human Immunology Team, Center for Infectious Diseases Education and Research,; 5 Laboratory of Experimental Immunology, WPI Immunology Frontier Research Center,; 6 Laboratory of Immune Regulation, WPI Immunology Frontier Research Center,; 7 Genome Information Research Center, Research Institute for Microbial Diseases,; 8 Single Cell Genomics, Human Immunology, WPI Immunology Frontier Research Center,; 9 Integrated Frontier Research for Medical Science Division, Institute for Open and Transdisciplinary Research Initiatives,; 10 Single Cell Immunology, Human Immunology, WPI Immunology Frontier Research Center,; 11 Center for Infectious Diseases Education and Research, and; 12 Statistical Immunology, WPI Immunology Frontier Research Center, Osaka University, Osaka, Japan.; 13 Laboratory for Systems Genetics, RIKEN Center for Integrative Medical Sciences, Yokohama, Japan.; 14 Japan Agency for Medical Research and Development – Core Research for Evolutional Science and Technology, Osaka University, Osaka, Japan.; 15 Center for Advanced Modalities and DDS, Osaka University, Osaka, Suita, Japan.

**Keywords:** COVID-19, Vaccines, Cellular immune response, Epigenetics, Innate immunity

## Abstract

Consecutive mRNA vaccinations against SARS-CoV-2 reinforced both innate and adaptive immune responses. However, it remains unclear whether the enhanced innate immune responses are mediated by epigenetic regulation and, if so, whether these effects persist. Using mass cytometry, RNA-Seq, and ATAC-Seq, we show that BNT162b2 mRNA vaccination upregulated antiviral and IFN-stimulated gene expression in monocytes with greater effects after the second vaccination than those after the first vaccination. Transcription factor–binding motif analysis also revealed enriched IFN regulatory factors and PU.1 motifs in accessible chromatin regions. Importantly, although consecutive BNT162b2 mRNA vaccinations boosted innate immune responses and caused epigenetic changes in isolated monocytes, we show that these effects occurred only transiently and disappeared 4 weeks after the second vaccination. Furthermore, single-cell RNA-Seq analysis revealed that a similar gene signature was impaired in the monocytes of unvaccinated patients with COVID-19 with acute respiratory distress syndrome. These results reinforce the importance of the innate immune response in the determination of COVID-19 severity but indicate that, unlike adaptive immunity, innate immunity is not unexpectedly sustained even after consecutive vaccination. This study, which focuses on innate immune memory, may provide novel insights into the vaccine development against infectious diseases.

## Graphical abstract

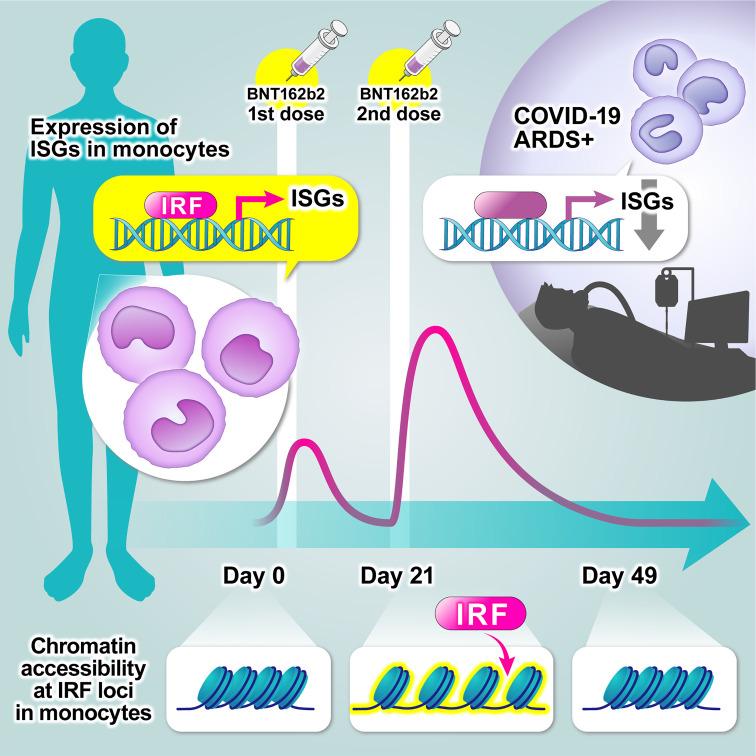

## Introduction

Since the start of the COVID-19 pandemic in December 2019, nearly 300 million people have been infected, and by the end of 2021, more than 5 million people had died. Over 40% of the world’s population has been vaccinated against SARS-CoV-2 as part of the effort to combat the COVID-19 pandemic. Among the available vaccines, mRNA-based vaccines that encode the SARS-CoV-2 spike protein, including the BNT162b2 vaccine (Pfizer-BioNTech) and mRNA-1273 vaccine (Moderna), were among the first administered and have shown significant efficacy (1).

SARS-CoV-2 mRNA vaccines induce the SARS-CoV-2 antigen–specific antibody and T cell responses (2–4). The boost in antibody production provided by revaccination with the BNT162b2 vaccine and the long-term persistence of memory B and T cell responses against SARS-CoV-2 indicate the formation of adaptive immune memory (3, 5). Interestingly, the innate immune response is also reinforced after the second vaccination, as shown by an observed increase in the frequency of CD14^+^CD16^+^ monocytes and enhancement of the IFN-stimulated gene (ISG) response in a myeloid cell cluster (CD14^+^BDCA1^+^PD-L1^+^ cells), which is a heterogeneous mix of classical monocytes (cMono), a classical DC subtype, and intermediate monocytes (intMono) (5). However, it is unclear whether the enhanced innate immune responses after the second vaccination are associated with innate immune memory, and if so, how long the memory persists in the immune cells.

Innate immune memory, also termed “trained immunity,” is an immunological memory that involves functional changes in innate immune cells after infection or vaccination, showing increased response to restimulation, which is similar to B and T cells’ adaptive immune memory (6–8). Trained immunity is characterized by epigenetic reprogramming in innate immune cells such as monocytes (9, 10) and NK cells (11) after infection or vaccination. The live-attenuated bacillus Calmette–Guérin (BCG) vaccine (9, 12), influenza vaccine (13, 14), and inactivated mucosal vaccine (15) induce epigenetic changes in monocytes, exerting protective effects against viral infections. Despite these findings, it is still unclear whether SARS-CoV-2 mRNA vaccines induce a similar alteration.

In this study, we reveal that the BNT162b2 mRNA vaccine induces epigenetic changes in monocytes after the first vaccination, which result in an enhanced type I IFN response at the time of the second vaccination. Importantly, however, we show that this effect is transient and diminishes 28 days after the second vaccination.

## Results

### The vaccinated cohort displays typical adaptive immune signatures and notable innate immune responses.

**Figure 1:**
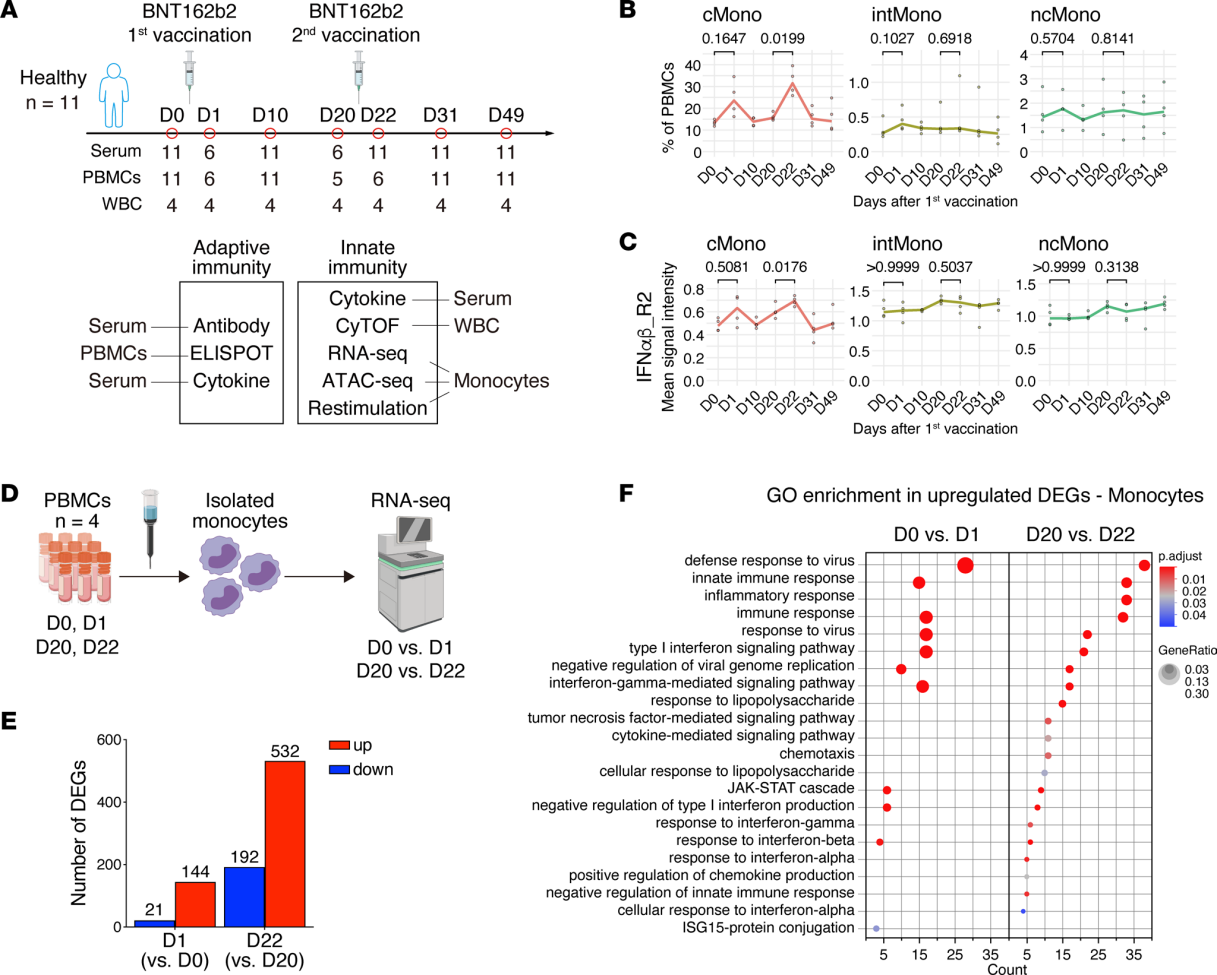
BNT162b2 vaccine alters the innate immune response in monocytes after the first immunization. (**A**) Study design and overview of the experiments. Healthy donors (*n* = 11) enrolled in this study received 2 doses of the BNT162b2 (30 μg/dose) vaccine at 3-week intervals. Blood samples were collected before (D0) and D1, D10, D20, D22, D31, and D49 days after the first vaccination. (**B** and **C**) Changes over time in the percentage of monocytes among PBMCs (**B**) and in the expression of IFNα/β-R2 on cMono, intMono, and ncMono (**C**). (**D**) Schematic overview of the RNA-Seq experiment performed using monocytes isolated from PBMCs collected from healthy individuals (*n* = 4) before and after vaccination (D0, D1, D20, and D22). (**E**) Numbers of DEGs in isolated monocytes on D1 and D22 compared with D0 and D20 (|log_2_ fold change| > 1 and *P*_adj_ < 0.05). (**F**) Gene Ontology (GO) enrichment analysis of upregulated DEGs in isolated monocytes after BNT162b2 vaccination. All the significantly enriched terms are listed (*P*_adj_ < 0.05). Left, comparison before (D0) and after (D1) the first vaccination; right, comparison before (D20) and after (D22) the second vaccination. The *x* axis shows number of genes included in each pathway. The dot color and size represent the statistical significance and the ratio of genes enriched in the pathway to the total enriched genes, respectively. GeneRatio shows the ratio of the number of genes included in each pathway to the total number of upregulated DEGs. WBCs, white blood cells; cMono, classical monocytes; intMono, intermediate monocytes; ncMono, nonclassical monocytes. Statistical analysis was performed using a repeated-measures 1-way ANOVA with a Greenhouse-Geisser correction and a Bonferroni post hoc test (**B** and **C**).

To investigate the immune response induced by the BNT162b2 mRNA vaccine, healthy health care workers with no history of SARS-CoV-2 infection and no previous vaccination against SARS-CoV-2 were enrolled (*n* = 11; all male; age range, 31–42 years) (Supplemental Table 1; supplemental material available online with this article; https://doi.org/10.1172/jci.insight.163347DS1). The participants received 2 doses of the BNT162b2 mRNA vaccine injected into the deltoid muscle at a 3-week interval (30 μg/dose, day 0 [D0] and D21). Blood samples were collected prior to the first vaccination (D0) and on D1, D10, D20, D22, D31, and D49 after the initial vaccination (Figure 1A).

To confirm that the results from studying this cohort would be applicable to the general situation among the wider population, previously demonstrated changes in the adaptive immune response were confirmed before evaluating the innate immune response: immunization with 2 doses of BNT162b2 significantly increased the levels of serum neutralizing antibodies and serum IgG, IgA, and IgM class antibodies, which bind to the spike protein and receptor-binding domain (RBD) regions of SARS-CoV-2 (Supplemental Figure 1, A and B), and the results of an IFN-γ ELISPOT assay indicate that immunization enhanced the T cell responses to SARS-CoV-2 spike protein peptides (Supplemental Figure 1C). These results indicate that the vaccination cohort participants responded to the vaccination as expected.

To further investigate the immune responses after the first and second vaccinations, we measured the serum levels of 21 cytokines and chemokines related to antiviral and inflammatory responses using a bead-based immunoassay before (D0) and after (D1 and D22) the vaccinations (Supplemental Figure 2A). We observed robust induction of IFN-γ and CXCL-10 in serum after the second vaccination compared with after the first vaccination (Supplemental Figure 2B). These cytokines and chemokines play an important role in promoting the innate immune responses involved in the effective development of humoral immunity after the boost immunization (16). Collectively, the enhanced immune response resulting from the boost immunization suggests that the innate immune responses may change after the initial vaccination.

### The type I IFN response of monocytes is more robust at the time of the second vaccination than at the time of the first.

We next investigated the effect of the vaccine on immune cell populations after the first and second vaccine doses with cytometry by TOF (CyTOF) using WBCs collected from 4 participants among those described in the previous subsection at all 7 time points described in the study design (Supplemental Figure 3A). Self-organizing map (FlowSOM) clustering of CyTOF data was used to classify the WBCs into 10 major immune subsets (Supplemental Figure 3B). In the peripheral blood mononuclear cell (PBMC) population, the proportion of myeloid cells (monocytes and DC) only showed an increasing trend on the day after the first and second vaccinations compared with the day before vaccination (Supplemental Figure 3C). We next classified monocytes into cMono, intMono, and nonclassical monocytes (ncMono) (Supplemental Figure 3D). The proportion of cMono was significantly more elevated after the second vaccination than after the first vaccination (Figure 1B). The expression levels of type I IFN receptor (IFNαβ_R2) on cMono were also more significantly upregulated on the day after the second dose than on the day after the first dose (Figure 1C). Additionally, the expression levels of Fcγ receptor 1 (FcγR1) and ICAM-1 (CD54), previously found to be induced on monocytes by IFNs or inflammatory cytokines (17, 18), were more highly expressed on monocytes after the second dose than after the first dose (Supplemental Figure 3, E and F). Collectively, these data demonstrate that the BNT162b2 mRNA vaccine stimulated monocytes more strongly after the second immunization than after the first immunization.

To evaluate the enhanced immune responses of monocytes in more detail, we performed transcriptome analysis of monocytes before and after the first (D0 versus D1) and second (D20 versus D22) vaccination (Figure 1D). The number of differentially expressed genes (DEGs) after the first vaccination (D1) relative to prevaccination (D0) was 165 (upregulated, 144; downregulated, 21), whereas the number of DEGs after the second vaccination (D22) was 724 (upregulated, 532; downregulated, 192) (Figure 1E). Gene Ontology (GO) enrichment analysis of the upregulated genes revealed many immune response terms common to the samples obtained after the first and second vaccinations (D1 and D22), such as antiviral and type I IFN responses (Figure 1F). These genes, including *CXCL10*, *IL6*, *FCGR1*, and *ICAM1*, were more significantly upregulated on D22 than on D1 (Supplemental Figure 4), consistent with the data from the bead-based immunoassay of serum and CyTOF analysis. These results indicate that the antiviral and type I IFN–specific innate immune transcriptional responses are enhanced in monocytes after BNT162b2 mRNA vaccination and that this response is more notable and widespread after the second vaccination than after the first vaccination.

### The BNT162b2 mRNA vaccine induces transient epigenetic changes in monocytes.

**Figure 2:**
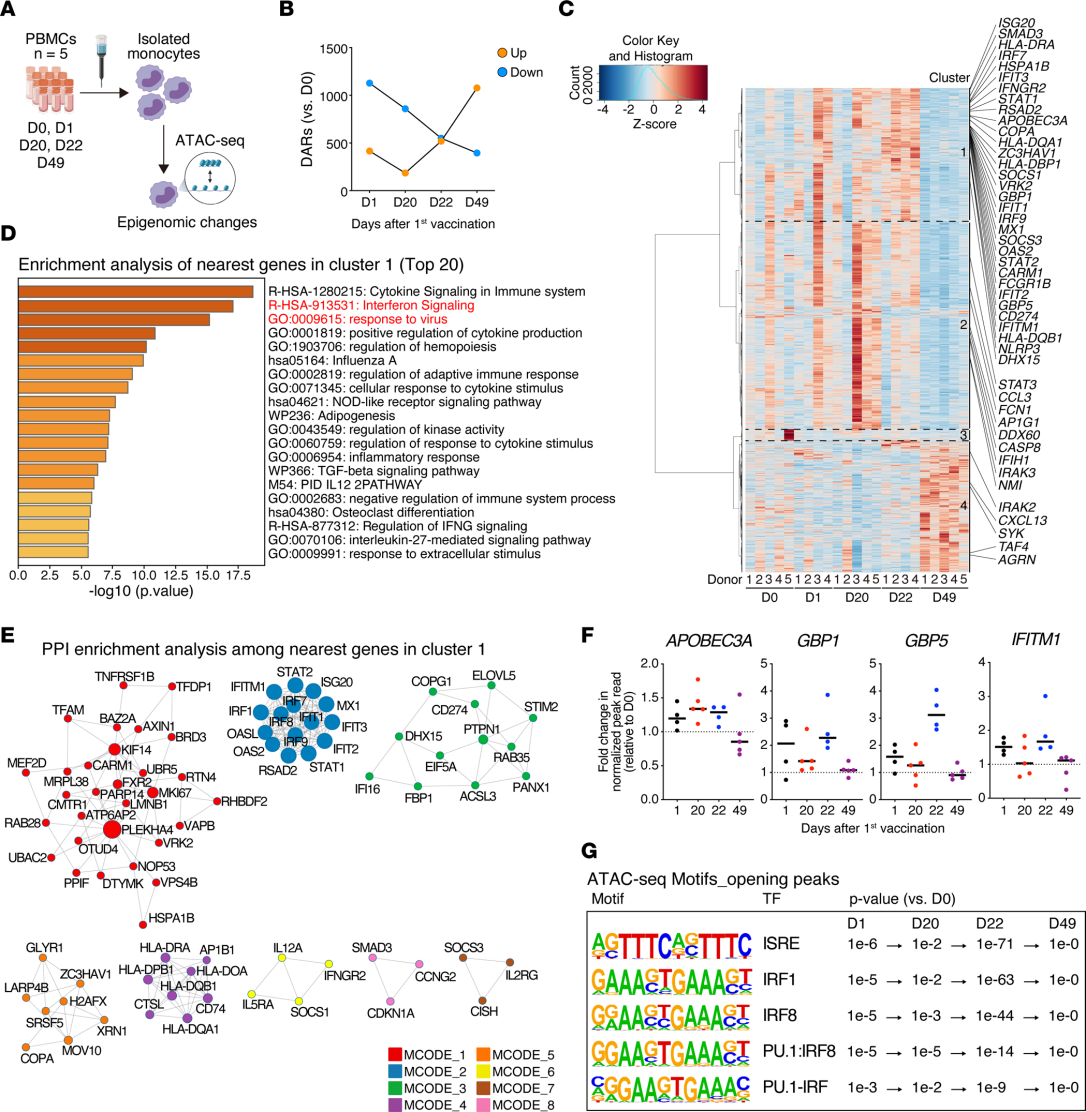
Epigenomic changes in monocytes regulate the innate immune responses to the BNT162b2 mRNA vaccine. (**A**) Schematic overview of the ATAC-Seq experiment of monocytes magnetically separated from PBMCs collected from healthy individuals before (D0, *n* = 5) and after (D1 and D22, *n* = 4; D20 and D49, *n* = 5) vaccination. All 4 individuals included in the RNA-Seq analysis (in Figure 1) were included in the ATAC-Seq analysis. (**B**) Numbers of differentially accessible chromatin regions (DARs) (|log_2_ fold change| > 1 and *P* < 0.05) in isolated monocytes on D1, D20, D22, and D49 compared with those on D0 were identified using edgeR (*n* = 5 per group). (**C**) Heatmap of *Z* scores of the normalized read counts identified by ATAC-Seq of isolated monocytes on D0, D1, D20, D22, and D49. Annotated genes were related to the innate immune responses among the nearest genes in each cluster. (**D**) Enrichment analysis of the nearest genes detected in cluster 1 as conducted with Metascape (http://metascape.org). The top 20 significantly enriched terms are listed (*P* < 0.05). Innate immune response terms are marked in red. (**E**) PPI network analysis among the nearest genes in cluster 1 using molecular complex detection algorithm as conducted with Metascape (http://metascape.org). The components of each molecular complex detection (MCODE) are listed in Supplemental Figure 5B. (**F**) Changes in normalized peak counts nearest antiviral and IFN-stimulated genes identified by ATAC-Seq of isolated monocytes. Fold changes are represented compared with D0. (**G**) Enriched known motifs identified using hypergeometric optimization of motif enrichment (HOMER) among enhanced chromatin accessibility regions on D1, D20, D22, and D49 compared with those on D0. TF, transcription factor.

To investigate whether the enhanced type I IFN signaling in monocytes observed after the second vaccination resulted from epigenetic reprogramming, we performed an assay for transposase-accessible chromatin with high-throughput sequencing (ATAC-Seq) to assess genome-wide chromatin accessibility (19). We were particularly interested in assessing whether any changes in monocytes persisted after the second vaccination. To perform this analysis, we isolated monocytes from the PBMCs obtained from 5 participants, including the 4 subjects for CyTOF and RNA-Seq analysis, before and after each vaccination dose (D0, D1, D20, D22, and D49) (Figure 2A) and detected 1,544; 1,045; 1,070; and 1,473 differentially accessible regions (DARs) on D1, D20, D22, and D49 compared with D0, respectively (Figure 2B). The hierarchical clustering of the normalized peak reads illustrated changes in the chromatin accessibility of monocytes after vaccination (Figure 2C). Enrichment analysis of all the nearest genes in a cluster showed terms related to innate immune responses in clusters 1 and 2, especially cluster 1 (Figure 2D and Supplemental Figure 5A). Additionally, protein-to-protein interaction (PPI) enrichment analysis among genes in cluster 1 revealed a densely connected network of immune responses, such as IFN signaling and response to virus (Figure 2E and Supplemental Figure 5B). The modules in MCODE2 showed a strong association with type I IFN–mediated antiviral responses: IFN regulatory factor (IRF) 7 is the master regulator of type I IFN production (20), and ISG factor 3 (STAT1, STAT2, and IRF9) activates downstream type I IFN signaling and induces the expression of antiviral effector ISGs (IFN induced transmembrane protein 1 [IFITM1], myxovirus resistance protein 1 [MX1], IFN-induced proteins with tetratricopeptide repeats 2 [IFIT2], IFIT3, and 2’-5’-oligoadenylate synthetase 2 [OAS2]) (21). In contrast, in clusters 3 and 4, the term and PPI networks related to innate immune responses were not detected (Supplemental Figure 5, C and D). Cluster 1 showed the chromatin accessibilities of several antiviral and ISGs that increased at D1, decreased at D20 but remained higher than those at D0, and increased again at D22 (Figure 2F). Interestingly, we observed a reduction in the chromatin accessibility at D49 back to the levels at D0 (Figure 2, C and F), indicating that these epigenomic changes did not persist.

Furthermore, hypergeometric optimization of motif enrichment (HOMER) motif searches among open peaks revealed that IFN-stimulated response element–like motifs were more accessible on D1, D20, and D22 and that transcription factor–binding (TF-binding) motifs associated with IRFs and PU.1 were enriched in monocytes at the same time points (Figure 2G). These TF-binding motifs, known to cooperate with each other to activate antiviral defense programs (22), were most significantly enriched after the second vaccination (D22) (Figure 2G). Notably, they were enriched in open peaks on D20 but not on D49 (Figure 2G). Additionally, PU.1 and multiple AP-1 member motifs, including Fosl2, Jun–AP-1, and JunB, were significantly detected in closing peaks on D49 according to HOMER analysis (Supplemental Figure 5E). These results suggest that epigenetic reprogramming in monocytes may be involved in the enhanced innate immune response seen after the second immunization. Additionally, they indicate that these primed immune responses, including the type I IFN response of monocytes, returned to prevaccination levels 28 days after the second immunization.

### Epigenetic changes in monocytes are linked to their functions.

**Figure 3:**
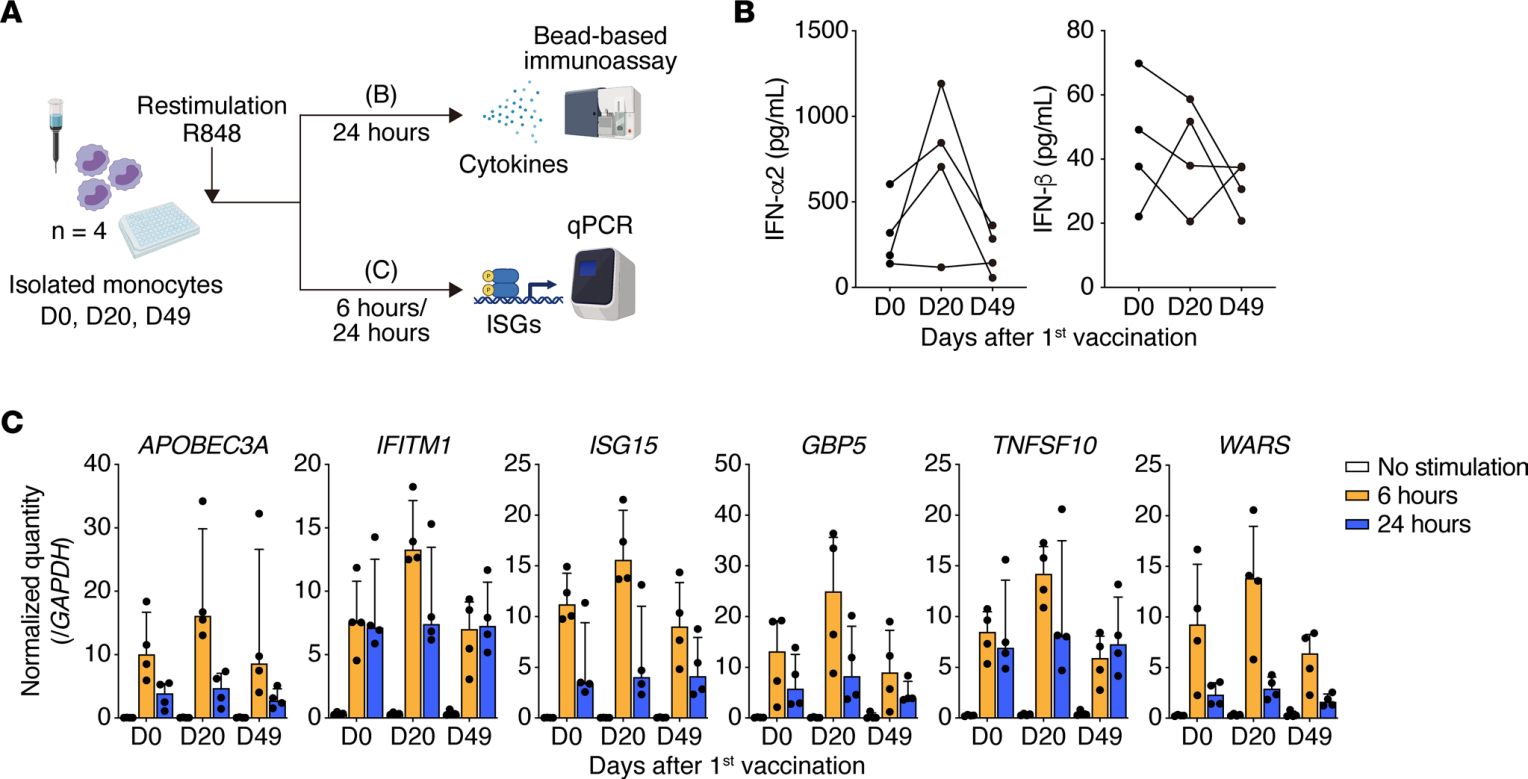
Dynamic changes in innate immune responses in monocytes after induction by the BNT162b2 mRNA vaccine. (**A**) Overview of the experiments. Restimulation of monocytes isolated from PBMCs on D0, D20, and D49 with R848 for 6 or 24 hours (*n* =4 per group). All 4 individuals included in the ATAC-Seq analysis (in Figure 2) were included in the restimulation experiment. R848 is a ligand for TLR7 and TLR8, which mimics single-stranded RNA viral pathogens. (**B**) Concentrations of type I IFNs (IFN-α2 and IFN-β) in the culture supernatant after stimulation of isolated monocytes with R848 (100 ng/mL) for 24 hours (D0, D20, and D49; *n* = 4 per group). Type I IFN levels were measured by a bead-based immunoassay. Each dot represents an individual. (**C**) Antiviral and IFN-stimulated gene (*APOBEC3A*, *IFITM1*, *ISG15*, *GBP5*, *TNFSF10*, and *WARS*) expression levels were quantified by qPCR before and after stimulation of isolated monocytes with R848 (100 ng/mL) for 6 and 24 hours (*n* = 4 per group). The gene expression levels were normalized to those of *GAPDH*. Each dot represents an individual. ISGs, IFN-stimulated genes.

To clarify the functional effects of the epigenetic changes on monocytes, we isolated monocytes from the PBMCs of participants prior to the first vaccination, at D20, and at D49 and stimulated the isolated monocytes with R848. R848 is a ligand for TLR7 and TLR8, which mimics viral pathogen–associated molecular patterns (such as single-stranded RNA, which is present during SARS-CoV-2 infection). After 24 hours of stimulation, we measured the levels of secreted cytokines in culture supernatants using bead-based immunoassays (Figure 3A). In the culture supernatant, the level of IFN-α2, a type I IFN, was elevated to a greater extent at D20 and returned almost to baseline at D49 (Figure 3B). Additionally, the same trend was observed for type II and III IFNs (IFN-γ and IFN-λ2/3, respectively) and proinflammatory cytokines (IL-1β, IL-6, TNF-α, and GM-CSF) (Supplemental Figure 6A), although not for IFN-β, another type I IFN (Figure 3B). These results revealed that the initial vaccination enhanced TLR7/8 responses in monocytes.

To investigate changes in the rapid type I IFN response before and after vaccination, we examined antiviral and ISG expression at multiple points. We stimulated monocytes isolated from the PBMCs of participants with R848 for 6 and 24 hours and quantitated the gene expression at each time point using real-time PCR. The target gene expression at 24 hours was not significantly different before and after vaccination; however, the rapid type I IFN response at 6 hours was stronger in monocytes on D20 (Figure 3C and Supplemental Figure 6B). In particular, *APOBEC3A*, *IFITM1*, *GBP1*, *GBP5*, and *FCGR1* responded rapidly in monocytes isolated on D20; however, these enhanced responses were attenuated in monocytes isolated on D49 (Figure 3C and Supplemental Figure 6B). These effects are consistent with the chromatin accessibility changes between D20 and D49 revealed by ATAC-Seq (Figure 2F). These results indicate that the initial vaccination with the mRNA vaccine induced epigenomic changes in monocytes and that the second vaccination increased the innate immune response, at least partly, via regulation of DNA accessibility at specific inflammation-related loci; however, these changes were not sustained at longer time points after the second vaccination.

### Type I IFN responses of monocytes are impaired in unvaccinated patients with severe COVID-19.

We showed that innate immune responses, particularly antiviral and IFN responses, were induced in monocytes by the BNT162b2 mRNA vaccine. However, the biological relevance of these innate immune responses to SARS-CoV-2 infection is unknown. We aimed to identify IFN signatures associated with COVID-19 severity using single-cell RNA-Seq (scRNA-Seq) analysis of unvaccinated patients with COVID-19 and to investigate the similarity between these gene clusters and those induced by BNT162b2.

**Figure 4:**
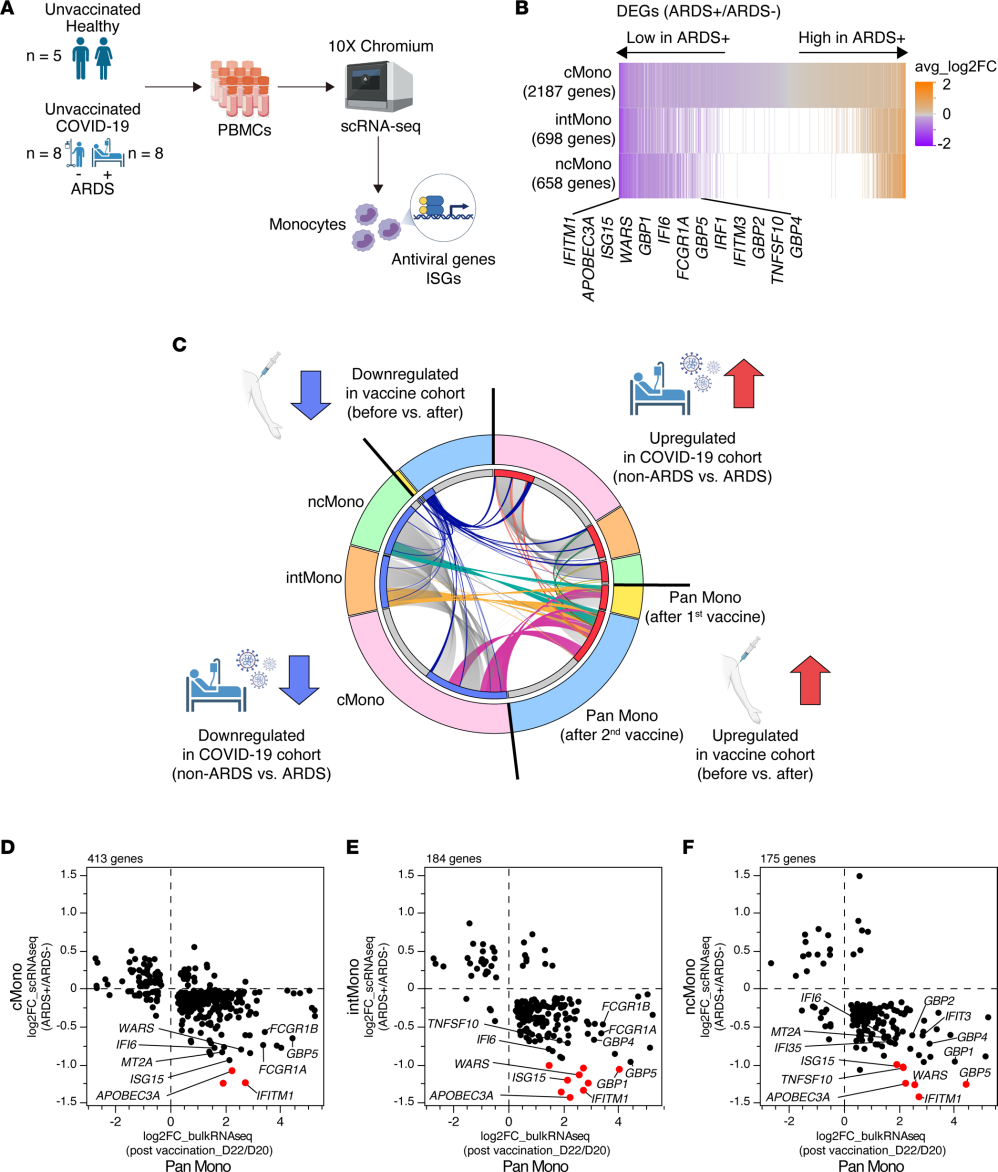
The strong relationship between the genes upregulated by vaccination and those downregulated during severe COVID-19. (**A**) Overview of scRNA-Seq experiment on PBMCs from patients with COVID-19 with and without ARDS (*n* = 8 per group) and from healthy donors (*n* = 5). No participants were vaccinated against SARS-CoV-2 infection. (**B**) DEGs in monocytes identified by scRNA-Seq analysis of patients with COVID-19 with and without ARDS. Selected ISGs are annotated. (**C**) Circos plot of the overlapping genes among DEGs in monocytes detected for the BNT162b2 mRNA vaccine cohort and the unvaccinated COVID-19 cohort (*P*_adj_ < 0.05). In the vaccine cohort, DEGs detected at D1 and D22 relative to D0 and D20, respectively, were analyzed. In the COVID-19 cohort, DEGs detected in monocytes of ARDS patients compared with non-ARDS patients were analyzed. Each segment of the outer circle represents a monocyte subclass (Pan Mono, pan monocytes; cMono, classical monocytes; intMono, intermediate monocytes; or ncMono, nonclassical monocytes) and gene expression pattern (upregulated or downregulated). The inner circle colored in red (upregulated genes) and blue (downregulated genes) represents the genes that are shared by multiple segments, and the gray circle represents genes that are unique to that segment. On the inside, each arc represents a gene list. The arcs linking the Pan Mono segment with the cMono, intMono, or ncMono segment are colored. (**D**, **E**, and **F**) Scatter plots showing the overlapping genes identified by Circos plot. The *y* axis represents DEGs in cMono (**D**), intMono (**E**), and ncMono (**F**) in the COVID-19 cohort (non-ARDS versus ARDS). The *x* axis represents DEGs in pan monocytes in the vaccine cohort (D20 versus D22). Genes with significantly changed expression are marked in red (|log_2_ fold change| > 1 and *P*_adj_ < 0.05 in both analyses). ARDS, acute respiratory distress syndrome; cMono, classical monocytes; intMono, intermediate monocytes; ncMono, nonclassical monocytes.

To explore the importance of the innate immune system in the prevention of severe disease, we analyzed the expression of genes associated with disease severity in the PBMCs of unvaccinated patients with COVID-19 with and without acute respiratory distress syndrome (ARDS) (*n* = 8 per group) and unvaccinated healthy donors (HDs) without SARS-CoV-2 infection (*n* = 5) (Figure 4A and Supplemental Table 2). After applying quality control filters and merging the samples, 119,293 high-quality single cells were obtained for the scRNA-Seq analysis and manually classified into 13 clusters (Supplemental Figure 7, A and B). A total of 19,289 single innate immune cells (myeloid cells, and plasmacytoid DCs [pDC]) were ultimately included and were classified into 5 clusters (cMono, intMono, ncMono, DC, and pDC) (Supplemental Figure 7, C and D). We detected 2187, 698, and 658 significantly DEGs between patients with ARDS-complicated COVID-19 and patients with non-ARDS COVID-19 in the cMono, intMono, and ncMono clusters, respectively. The expression of antiviral genes and ISGs was upregulated in patients with COVID-19 compared with that in the HDs (Supplemental Figure 7E); however, these expression levels in patients with ARDS were downregulated compared with those in non-ARDS patients (Figure 4B), indicating that an impaired type I IFN response to SARS-CoV-2 infection is associated with severe COVID-19, consistent with previous reports (23–25).

### The innate immunity-related genes downregulated during severe COVID-19 are associated with those upregulated by mRNA vaccination.

Next, we investigated the similarities between the gene signatures associated with COVID-19 severity and those induced by the BNT162b2 mRNA vaccine, as revealed in our vaccine cohort study described above. A total of 413 (22.7%), 184 (10.1%), and 175 (9.62%) genes among the DEGs (1,820 genes) identified in the monocytes from the vaccine cohort were also present among the DEGs observed in the cMono, intMono, and ncMono in the COVID-19 cohort, respectively (Supplemental Figure 7F). Interestingly, the genes upregulated by BNT162b2 showed strong commonality with those downregulated in monocytes of ARDS patients with COVID-19, while the genes downregulated by BNT162b2 shared little overlap with those in the monocytes of patients with COVID-19 (Figure 4C). In particular, several genes that play an important role in antiviral responses, such as *APOBEC3A* (26, 27), *GBP* family genes (28, 29), and *IFITM* family genes (30, 31), were identified in the shared gene set (Figure 4, D–F). Indeed, enrichment analysis of DEGs in monocytes revealed that innate immune responses, such as response to virus and IFN signaling, were enriched for up- and downregulated genes in the vaccine cohort and the COVID-19 cohort, respectively (Supplemental Figure 7G). These findings suggest that the innate immune responses of monocytes induced by BNT162b2 mRNA may be involved in the prevention of COVID-19 severity.

## Discussion

In this study, consecutive vaccination with BNT162b2 mRNA was found to enhance both adaptive and innate immune responses; however, the enhancement of innate immunity was not sustained. The genes that upregulated after vaccination were strongly related to those downregulated during severe COVID-19 infection.

Early in the COVID-19 pandemic, it was hypothesized that long-term enhancement of the innate immune response, known as “trained immunity,” via the epigenetic reprogramming of innate immune cells after BCG vaccination may contribute to the prevention of SARS-CoV-2 infection and severity (32). In this study, ATAC-Seq of monocytes showed increased accessibility of chromatin regions associated with ISG expression after the second vaccination, but these changes disappeared after only 4 weeks. Thus, this observation may indicate innate immune cells displaying a form of “short-term epigenetic memory” (33, 34). In particular, we observed a transient enhancement of type I IFN responses after vaccinations. Type I IFNs play an important role not only in the host defense against viruses (35), but also in the development and maintenance of adaptive immunity, including B and T cell responses (36, 37). Regarding the humoral immune response, type I IFNs directly activate B cells, promoting antibody production during viral infections (38). Similarly to the effect observed during viral infection, the enhanced innate immune responses resulting from mRNA vaccination are associated with the production of neutralizing antibodies specific to SARS-CoV-2 (5). On the other hand, persistent type I IFN production may lead to failure to maintain higher antibody titers (39) or improper autoantibody production, resulting in autoimmune disease (40). Although the enrichment analysis on the gene set in cluster 4 of ATAC-Seq showed no significant pathways associated with immune responses, chromatin accessibility at the *CXCL13* locus was still increased 28 days after the second vaccination, suggesting that the innate immune memory induced by mRNA vaccines may promote B cell maturation and antibody responses and help develop a rapid host defense against natural infection (41, 42). Taken together, these results indicate that the innate immunity induced by mRNA vaccines may not be directly involved in the long-term prevention of SARS-CoV-2 infection but, instead, play an important role in driving adaptive immunity, including the production of antigen-specific antibodies. However, there is insufficient evidence to explain if the short-term innate immune memory induced by mRNA vaccines remains; thus, further research is needed to address this question.

To investigate the factors contributing to the severity of COVID-19, we assessed the role of innate immunity in severe disease using a cohort of unvaccinated patients with COVID-19. In this unvaccinated cohort, the expression of ISGs in monocytes was markedly reduced in patients presenting with ARDS. Type I IFN is an important cytokine for the activation of Th1 and NK cells, which function to eliminate virus-infected cells (43). Indeed, impaired type I IFN responses (23) and preexisting autoantibodies against type I IFN (44, 45) have been shown to be associated with the severity of COVID-19. On the other hand, it has also been reported that hypoxemia complicated with ARDS causes monocytopenia and suppression of type I IFN signaling in mice (46) and that SARS-CoV-2 ORF6 (47) and nucleocapsid proteins (48) suppress type I IFN signaling in vitro. These conflicting reports suggest that the lower type I IFN responses to SARS-CoV-2 may be both a cause and a consequence of severe COVID-19. In any event, type I IFN signaling is essential as a viral restriction mechanism; its successful response may help limit the occurrence of other risk factors for severe COVID-19 such as cytokine storm (49). Interestingly, the gene set downregulated in patients with COVID-19 with ARDS was similar to the gene set strongly induced in monocytes by the BNT162b2 mRNA vaccine; this means that the innate immune responses induced by mRNA vaccines may compensate for the decreased IFN responses in patients with severe COVID-19. Therefore, to ensure the prevention of severe COVID-19 after a second or third vaccination dose, activating and sustaining innate immune responses, including the IFN response, may be important. Further investigations are needed to assess not only the dynamics of neutralizing antibody titers, but also the effects on innate immunity, such as the IFN response, to determine the optimal dosing intervals for mRNA vaccines.

It is important to take into consideration interactions between innate and adaptive immune responses. In fact, polyfunctional CD4^+^ T cells producing IL-2, IFN-γ, TNF-α, and/or IL-4 remained abundant in the peripheral blood immediately before the second vaccination (5). In mice, the first dose of the BNT162b2 vaccine induced the remaining antigen-specific tissue-resident T cells to produce IFN-γ, TNF-α, and IL-2 in the lung or spleen immediately before the second vaccination (50). It is possible that these differences in adaptive immunity could be responsible for the epigenomic changes and the enhancement of innate immune responses in monocytes just after the second vaccination. However, despite these changes in adaptive immunity observed after the second vaccination (D42) (5), we could not detect an increase in chromatin accessibility in monocytes involved in the innate immune responses at D49, as described above. Further investigation is warranted to reveal the involvement of persistent epigenetic changes (13, 51) in the systemic immune responses to an mRNA vaccine.

Our report has the following limitations. First, the sample size was small; therefore, individual effects may have affected the analysis results. Second, all participants were male, and sex was, therefore, not considered in our study. Females have a stronger humoral immune response to the vaccine than males, and higher antibody titers are reportedly induced by BNT162b2 in females (52). Additionally, the IFN-α2 plasma levels induced by BNT162b2 are likely higher in females than in males (5). Further investigations are warranted to understand the impact of these differences in immune responses between females and males on epigenetic reprogramming in innate immune cells. Third, this study was a short-term observation, extending 49 days after the first immunization. It has been reported that the effectiveness of the BNT162b2 mRNA vaccine against SARS-CoV-2 infection gradually decreases after the second vaccination (53) and that a third dose of mRNA vaccine improves the protection against SARS-CoV-2 infection and severe COVID-19 due to enhanced adaptive immune responses (54, 55). However, it is unclear how the epigenomics of the innate immune system changes during subsequent vaccination and whether this contributes to the booster effect; therefore, further longitudinal observation will be needed. Forth, our transcriptional and epigenomic analyses were not performed at a single-cell level. A previous report has shown that the responses to the BNT162b2 mRNA vaccine vary between monocytes subsets (5). In our study, whether the similar gene signatures found between the vaccine cohort and COVID-19 cohort are shared among the same monocyte subsets remains an open question. Finally, we only analyzed circulating monocytes. Long-lasting innate memory is observed in tissue-resident macrophages (8). In our study, it remains unclear whether the mRNA vaccines act on tissue-resident macrophages, resulting in long-term innate immune memory.

We provide evidence of activating alterations in innate immune response-related genes with epigenetic changes in monocytes induced by the BNT162b2 mRNA vaccine and reveal that these effects are transient (Supplemental Figure 8). In the future, considering the importance of innate immune responses against viral infections, clarifying the long-term effects of consecutive mRNA vaccine doses on innate immune memory may lead to new strategies to combat viral infections.

## Methods

### Research participants.

We recruited participants into 2 cohorts. All participants were enrolled after providing written informed consent.

For the first cohort, to evaluate the immune response induced by the BNT162b2 mRNA vaccine, participants were recruited from March 15, 2021, to June 7, 2021, at Osaka University Hospital. All the participants were healthy health care workers with no history of SARS-CoV-2 infection and no previous vaccination against SARS-CoV-2 (*n* = 11; all male; age range, 31–42 years). We collected serum, PBMCs, and WBCs from them on D0, D1, D10, D20, D22, D31, and D49. These volunteers were then immunized with 2 doses (30 μg/dose) of the mRNA vaccine at 3-week intervals according to the normal protocols of the facility.

For the second cohort, to analyze the single-cell transcriptome of peripheral blood cells, we included unvaccinated patients with COVID-19 with ARDS (*n* = 8) or without ARDS (*n* = 8) who were admitted to Osaka University Medical Hospital between December 2019 and October 2021 and unvaccinated healthy donors (*n* = 5) different from the first cohort. We collected PBMCs from patients with COVID-19 when they were admitted to Osaka University Medical Hospital. For the patients with COVID-19 with ARDS, PBMCs were collected during ARDS status.

### Isolation and storage of serum and PBMCs.

For serum collection, peripheral blood samples were allowed to stand for 15 minutes at room temperature, centrifuged at 1000*g* for 15 minutes, and stored at −80°C until use. PBMCs were isolated from fresh whole blood collected in heparin-coated tubes by density gradient centrifugation using Leucosep (Greiner Bio-One, 227288-013) according to the instruction manual, and isolated PBMCs were stored in CELLBANKER cell freezing medium (Nippon Zenyaku Kogyo Co. Ltd., 11910) at −80°C until use.

### Serum SARS-CoV-2 neutralizing antibody quantification.

The concentration of serum SARS-CoV-2 NAb was measured using an iFlash3000 (Shenzhen Yhlo Biotech Co. Ltd., YH-C6111) fully automated chemiluminescence immunoassay (CLIA) analyzer and an iFlash-2019-nCoV NAb Kit (Shenzhen Yhlo Biotech Co. Ltd., YH-C86109) according to the manufacturer’s protocols. This kit consists of a 1-step competitive assay to measure the binding inhibition activity by antibodies against the RBD of SARS-CoV-2 binding to angiotensin-converting enzyme 2 (ACE2), which serves as a receptor for viral entry. The 4-point calibrator in this kit was used to generate a calibration curve. The results are reported as inhibitory activity in AU/mL, and the cutoff value was set at 10.0 AU/mL (≥10 AU/mL, positive; <10 AU/mL, negative). All the above processes were automated, and the results were obtained in a high-throughput manner by simply loading 150 μL of each serum sample into the iFlash3000.

### Serum SARS-CoV-2 antigen–specific antibody measurement.

IgG, IgA, and IgM against the SARS-CoV-2 spike protein subunits S1 and S2 and the spike RBD in serum collected from vaccinated participants (*n* = 11) were quantified by a multiplex bead-binding assay using Milliplex technology (HC19SERM1-85K, HC19SERG1-85K, HC19SERA1-85K; MilliporeSigma) following the manufacturer’s instructions. In brief, 25 μL of diluted serum sample (1:100) was mixed with 25 μL of bead mixture (3 antigen-immobilized beads and 3 control beads) in a 96-well plate containing 25 μL of assay buffer per well. After incubation on a plate shaker (600 rpm) for 2 hours at room temperature, the plates were washed 3 times with wash buffer using a Handheld Magnetic Microplate Washer (Bio-Rad), and 50 μL of PE-conjugated anti–human IgG, IgA, or IgM was added to each well. After incubation on a plate shaker (600 rpm) for 90 minutes, the plates were washed 3 times with wash buffer as described above, and then sheath fluid was added to each well for reading on a Bio-Plex 200 System (Bio-Rad). Fifty events per bead were counted, and median fluorescence intensity (MFI) data were analyzed with Bio-Plex Manager Software with default parameters (Bio-Rad) (version 6.2). The MFIs of the background wells were subtracted from the MFIs of the sample wells. The results were evaluated relative to the degree of increase in the MFIs compared with the MFIs before vaccination.

### ELISPOT assay.

SARS-CoV-2 antigen–specific T cell responses were assessed using a human IFN-γ/IL-4 Double-Color FluoroSpot assay (CTL, hT2003F) according to the manufacturer’s instructions. Briefly, 150,000 PBMCs/well collected from vaccinated participants (*n* = 11) were incubated with 2.0 μg/mL SARS-CoV-2 S1 scanning pools (MABTECH, 3629-1) for 48 hours at 37°C and in 5% CO_2_. The plates were analyzed using an ImmunoSpot S6 MACRO Analyzer (CTL), and the number of spot-forming units (SFU) was automatically calculated using ImmunoCapture software (version 7.0.7.3). The number of spots in negative control wells was subtracted from the number of spots in test wells, and the results are displayed as SFU in the Supplemental Figure 1C.

### Serum cytokine measurements.

Cytokines in serum obtained from vaccinated participants (*n* = 11) were quantified using a LEGENDplex Human Anti-Virus Response Panel (13-plex) (BioLegend, 740390) and a Human B cell Panel (13-plex) (BioLegend, 740527) according to the default protocols. In brief, the samples and standards were incubated with premixed beads on a plate shaker (800 rpm) for 2 hours at room temperature. After washing with 1× wash buffer, they were reacted with a detection antibody on a plate shaker (800 rpm) for 1 hour at room temperature, and then PE-conjugated beads were added and reacted on a plate shaker (800 rpm) for 30 minutes at room temperature. After washing with 1× wash buffer, the samples were read on a flow cytometer using a FACSCanto II (BD Biosciences). The Flow Cytometry Standard (FCS) files were analyzed using the LEGENDplex Data Analysis Software Suite (BioLegend), an online cloud-based program (https://legendplex.qognit.com/).

### Immune profiling of peripheral whole blood using mass cytometry.

A Maxpar Direct Immune Profiling Assay (Fluidigm, 201325) was used for immune profiling of peripheral whole blood according to the manufacturer’s protocol. In addition to the 30 premixed marker antibodies, antibodies specific for 13 markers, IFN-α/βR2, IFN-α/βR1, neuropilin-1, ICAM-1, Fcγ-RI, CXCR1, FCAR, CD147, CD44, semaphorin 4D (Sema4D), Sema6D, CXCR2, and PD-L1, were added (Supplemental Table 3). In brief, whole blood was incubated with a 100 U/mL heparin solution (Sigma Aldrich, H3149) to reduce nonspecific antibody binding at room temperature. After 20 minutes, aliquots consisting of 270 μL of whole blood and 3 μL of each additional antibody were placed in heparin-coated tubes containing a dry antibody pellet and incubated for 30 minutes at room temperature. After staining, RBCs were lysed with 250 μL of Cal-Lyse lysing solution (Invitrogen, GAS010S100) and 3 mL of Maxpar Water (Fluidigm) for 10 minutes each at room temperature. After lysis, the cells were washed 3 times with Cell Staining Buffer (CSB) (Fluidigm). Then, the cells were fixed with 1 mL of 1.6% paraformaldehyde for 10 minutes at room temperature and incubated with 125 nM Cell-ID Intercalator-Ir dissolved in Maxpar Fix and Perm Buffer (Fluidigm). After incubation for 30 minutes at room temperature, the cells were washed with CSB and Cell Acquisition Solution (CAS) (Fluidigm) and resuspended in CAS containing 0.1× EQ Four Element Calibration Beads at a cell concentration of 1 × 10^6^ cells/mL. After filtering the cells through a 35 μm cell strainer, data were acquired using Helios (Fluidigm). The acquired FCS files were normalized with CyTOF Software (version 7.0.8493).

### Mass cytometry data analysis.

Mass cytometry data analysis was conducted in R (version 4.0.3) using the following packages. FlowSOM clustering and uniform manifold approximation and projection (UMAP) were performed using Catalyst (version 1.14.1). WBC-level clustering was performed using the markers CD3, CD4, CD8a, CD19, CD14, CD16, CD11c, CD56, CD161, CD123, TCRγδ, CD66b, HLA-DR, FcγR1_CD64, and CD294. To exclude DCs from monocytes, clustering was performed using CD11c, CD14, HLA-DR, CD16, FCAR_CD89, CD38, CD294, CD45, ICAM-1_CD54, CD45RA, CD45RO, CD147, FcγR1_CD64, and Sema4D. Monocyte clustering was performed using CD11c, CD14, CD16, HLA-DR, CD38, CD45RA, CD45RO, FcγR1_CD64, PD-L1, and ICAM-1_CD54. All plots were generated using ggplot2 (version 3.3.5). Statistical tests were performed using rstatix (version 0.7.0). The R script is available on Github (https://github.com/jonasns/tmVac; ff3e517ee3e530c5dafad6b0d4642075304987f2). 

### Monocyte isolation.

Monocytes were isolated from frozen PBMCs collected from participants in the vaccine study by an indirect magnetic labeling system using a Pan Monocyte Isolation Kit (Miltenyi Biotec, 130-096-537) following the manufacturer’s protocol. This kit is capable of simultaneously isolating cMono (CD14^++^CD16^–^), intMono (CD14^++^CD16^+^), and ncMono (CD14^+^CD16^++^). In brief, frozen PBMCs were thawed and washed twice with RPMI 1640 (NACALAI TESQUE, 30264-85) supplemented with 10% FBS (Thermo Fisher Scientific, 10500064). After resuspension in PBS supplemented with 2 mM EDTA and 0.5% BSA, the PBMCs were incubated at 4°C for 5 minutes with FcR Blocking Reagent and Biotin-Antibody Cocktail, a cocktail of monoclonal anti-human antibodies against antigens that are not expressed on human monocytes. After the reaction, anti-biotin microbeads were added to the PBMCs and allowed to react for 10 minutes at 4°C. Unlabeled monocytes were collected by depletion of the magnetically labeled cells using a MACS cell separation system (Miltenyi Biotec). The purity of the isolated monocytes was assessed by a flow cytometer using a FACSCanto II flow cytometer (BD Biosciences). Isolated monocytes were used for the analysis of RNA-Seq (*n* = 4), ATAC-Seq (*n* = 5), and the restimulation experiment (*n* = 4) as described below.

### RNA-Seq of isolated monocytes.

Total RNA was extracted from the monocytes isolated from PBMCs (D0, D1, D20, and D22) as described above using a miRNeasy Mini Kit (QIAGEN, 217004) according to the manufacturer’s protocol. In brief, isolated monocytes were homogenized with QIAzol Lysis Reagent (QIAGEN, 79306), followed by separation of the aqueous phase using chloroform. The separated sample was mixed with 1.5 volumes of 100% ethanol, and the mixture was transferred into an RNeasy Mini spin column. After washing the column with Buffer RWT and Buffer RPE in turn, total RNA was eluted from the column by RNase-free water. The quality and quantity were assessed using an Agilent2100 bioanalyzer (Agilent Technologies). Full-length cDNA was generated using a SMART-Seq HT Kit (Takara Bio, 634436) according to the manufacturer’s instructions. An Illumina library was prepared using a Nextera XT DNA Library Preparation Kit (Illumina, FC-131-1096), according to the SMARTer Kit instructions. Sequencing was performed on an Illumina NovaSeq 6000 sequencer (Illumina) in 101 bp–end mode.

### RNA-Seq preprocessing.

The quality of raw paired-end sequencing reads was assessed using FastQC (56) with default parameters (version 0.11.7; https://www.bioinformatics.babraham.ac.uk/projects/fastqc/). Low-quality (<20) bases and adaptor sequences were trimmed with Trimmomatic software (57) (version 0.38) with the following parameters: ILLUMINACLIP: path/to/adapter.fa:2:30:10 LEADING:20 TRAILING:20 SLIDINGWINDOW:4:15 MINLEN:36. The trimmed reads were aligned to the hg38 reference genome using the RNA-Seq aligner HISAT2 (58) (version 2.1.0). The HISAT2-resultant .sam files were converted into .bam files with Samtools (59) and used to estimate the abundance of uniquely mapped reads with featureCounts (60) (version 1.6.3). The raw counts were normalized to transcripts per million (TPM) (61).

### RNA-Seq data analysis.

Based on the normalized counts, we performed comparison analyses among samples by hierarchical clustering, principal component analysis (PCA), Pearson’s correlation coefficient analysis, and scatter plot and heatmap construction. A tree diagram was produced by hierarchical clustering with the Wald method using each pair of Euclidean distances. A 2D plane based on the first and second principal components was created by PCA. The Pearson’s correlation coefficients of each gene were calculated, and scatter plots of comparisons between samples were constructed. Using the stats (version 3.6.1) and gplots (version 3.0.1.1) R packages, a heatmap was created by calculating the *Z* scores of the TPMs for each gene. Analysis of the differential gene expression in monocytes was performed on samples collected immediately before and after vaccination (D1 versus D0 and D22 versus D20). DEGs in monocytes were detected using DESeq2 (62, 63) (version 1.24.0) with the thresholds of |log_2_ fold change| > 1 and an adjusted *P* value (*P*_adj_) < 0.05 calculated by the Benjamini and Hochberg (BH) method (64). GO terms with *P*_adj_ < 0.05 determined by the BH method were extracted with DAVID (65, 66) (version 1.22.0).

### ATAC-Seq of isolated monocytes.

Samples for ATAC-Seq analysis were prepared from monocytes isolated from PBMCs (D0, D1, D20, D22, and d49) as described above. Libraries were prepared using an ATAC-Seq Kit (Active Motif, 53150) according to the manual. In brief, 100,000 cells were washed with ice-cold PBS and resuspended in ice-cold ATAC lysis buffer. After centrifugation at 500*g* for 10 minutes at 4°C, the pellets were resuspended with Tagmentation Master Mix and incubated at 37°C for 30 minutes in a thermostatic mixer (800 rpm). After incubation, the samples were mixed with DNA Purification Binding Buffer and 3M sodium acetate and were transferred to a DNA purification column. After washing the column with wash buffer containing 80% ethanol, the samples were eluted with DNA Purification Elution Buffer. Following purification, we performed PCR amplification of tagmented DNA using 25 μM i7/i5 Indexed Primers, 10 mM dNTPs, 5× Q5 Reaction Buffer, and 2 U/μL Q5 Polymerase under the following PCR conditions: 72°C for 5 minutes; 98°C for 30 seconds; and 10 cycles of 98°C for 10 seconds, 63°C for 30 seconds, and 72°C for 1 minute. After PCR amplification, the samples were purified using SPRI bead solution and eluted with 20 μL of DNA Purification Elution Buffer. The library quality and quantity were checked using a LabChip GX Touch HT (PerkinElmer) with an HT NGS 3K Reagent Kit (PerkinElmer), and the fragment size distribution was measured using quantitative PCR (qPCR) with a KAPA Library Quantification Kit (Kapa Biosystems). Sequencing was performed on an Illumina NovaSeq 6000 sequencer (Illumina) in 150-base paired-end mode.

### ATAC-Seq preprocessing.

FastQC (56) (version 0.11.5; https://www.bioinformatics.babraham.ac.uk/projects/fastqc/) was used to check the overall sequence data quality with default parameters. Low-quality (<20) bases and adapter sequences were trimmed by Trimmomatic software (57) (version 0.38) with the following parameters: ILLUMINACLIP: /path/to/adapter.fa:2:30:10 LEADING:20 TRAILING:20 SLIDINGWINDOW:4:15 MINLEN:36 Bowtie2 (67) (version 2.3.4.2) was applied to align the data to the hg38 human reference genome using the “-N 1 -X 2000 -U /path/to/sample_1_fq.gz,/path/to/sample_2_fq.gz” options. Samtools (58) was used to sort and remove the reads mapped to blacklisted regions of signal artifacts (68). The Samtools-resultant.bam files were used to perform peak calling with MACS2 software (69) using the “-q 0.01–nomodel” options. The peaks of all samples were merged with “bedtools merge” (70). The abundance of the mapped reads was estimated with featureCounts (60) (version 1.6.3). The raw read counts were normalized to the trimmed mean of M values (TMM).

### ATAC-Seq data analysis.

Based on the normalized counts, we conducted comparative analyses of all samples by hierarchical clustering, PCA, correlation analysis, and heatmap construction. Hierarchical clustering by the Wald method using each pair of Euclidean distances resulted in a tree diagram. Each sample was projected onto a 2D plane of the first and second PCA axes. Scatter plots were constructed by calculating Pearson’s correlation coefficients of each peak between a pair of samples. Heatmaps were created by calculating *Z* scores of the count data using the stats (version 3.6.1) and gplots (version 3.0.1.1) R packages. We then compared groups of samples to detect differential peak regions using the edgeR package (71) (version 1.24.0) with the threshold of |log_2_ fold change| > 1 and *P* < 0.05. The nearest genes from each peak were annotated by the ChIPpeakAnno R package (72). Gene enrichment analysis of the nearest genes was performed using the Metascape online platform (73). Motif analysis of the differential peak regions was conducted with HOMER software (74).

### Restimulation of isolated monocytes and quantification of cytokines in supernatants.

After isolating monocytes from PBMCs collected before (D0) and after (D20 and D49) vaccination as described above, 1 × 10^5^ cells per well were cultured with RPMI 1640 (NACALAI TESQUE, 30264-85) and 100 ng/mL R848 (InvivoGen, tlrl-r848) in 96-well flat-bottom plates (Nunc) for 6 hours or 24 hours at 37°C and in 5% CO_2_. After centrifugation at 300*g* for 5 minutes, the supernatant was transferred to another plate to perform a cytokine quantification assay, and the cells were resuspended in 350 μL of buffer RL containing 143 mM 2-mercaptoethanol (NIPPON Genetics Co. Ltd., FG-81250) for RNA extraction and subsequent qPCR. These samples were stored at −80°C until use. Cytokines in the supernatant were quantified using a LEGENDplex Human Anti-Virus Response Panel (13-plex) (BioLegend, 740390) as described above for serum cytokine quantification.

### RNA extraction and qPCR for isolated monocyte restimulation.

RNA was extracted from monocytes stimulated with R848 as described above using a FastGene RNA Premium Kit (NIPPON Genetics Co. Ltd., FG-81250) and was reverse transcribed with SuperScript IV VILO Master Mix (Invitrogen, 11766500) following the manufacturer instructions. qPCR was performed using PowerTrack SYBR Green Master Mix (Applied Biosystems, A46109) with a QuantStudio 7 system (Thermo Fisher Scientific). For all samples, we determined the target quantity from a standard curve and normalized it to *GAPDH*. The primer sequences are listed in Supplemental Table 4.

### Preprocessing and scRNA-Seq of samples from patients with COVID-19.

PBMCs for scRNA-Seq were obtained from patients with COVID-19 (ARDS complicated, *n* = 8; non-ARDS complicated, *n* = 8) and healthy donors (*n* = 5) as described above. Single-cell suspensions were loaded on a 10x Genomics Chromium Controller according to the manufacturer’s instructions for Chromium Single Cell V(D)J Reagent Kits (v1.1 Chemistry), using a Chromium Next GEM Single Cell 5′ Library & Gel Bead Kit v1.1 (10x Genomics, PN-1000167), Chromium Next GEM Chip G Single Cell Kit (10x Genomics, PN-1000127), and Single Index Kit T Set A (10x Genomics, PN-1000213). Following gel beads-in-emulsion (GEM) generation, cDNA with a cell barcode and unique molecular index (UMI) was produced from each encapsulated cell in an oil droplet by reverse transcription. After amplification of cDNA, the fragmentation, end repair, and polyA tagging steps were performed. The libraries were sequenced on an Illumina NovaSeq 6000 in paired-end mode (read 1, 26 bp; read 2, 91 bp). The libraries were processed using Cell Ranger 5.0.0 (10x Genomics). Count matrices were constructed using dropEst (75) from BAM files obtained by alignment with STAR (76) using the GRCh38 human reference genome. Cells with less than 1,000 UMIs or more than 20,000 UMIs, and cells containing more than 10% reads from mitochondrial or hemoglobin genes, were filtered. Furthermore, for each sample, doublets identified by Scrublet (77) were removed.

### scRNA-Seq data analysis.

The Seurat package in R (v3.2.2) was used to perform scaling, transformation, clustering, dimensionality reduction, differential expression analysis, and visualization (78). The SCTransform function was used to scale and transform the data, and linear regression was used to remove unwanted variability according to the percentage of mitochondrial reads. We identified “anchors” using the FindIntegrationAnchors function between individual data sets based on 3,000 shared highly variable genes (HVGs), which were identified using the SelectIntegrationFeatures function, and then we created a batch-corrected expression matrix of all cells by applying these anchors to the IntegrateData function. We performed PCA and UMAP dimension reduction with 30 principal components (79). We used the FindNeighbors function to calculate a nearest-neighbor graph using the 30 dimensions of the PCA reduction and then used the FindClusters function for clustering. We determined the cellular identity by finding DEGs for each cluster using the FindMarkers function with the parameter “test.use=wilcox”, and we obtained 13 cell clusters by comparing those markers to known cell type–specific genes. To analyze the subcluster of innate immune cells including myeloid cells, we extracted and reintegrated CD14^+^ monocytes, CD16^+^ monocytes, DCs, and pDCs, following the procedure described above, except that 2,000 shared HVGs were used. After integration, clustering and cluster annotation were performed as described above, and 5 cell clusters were obtained. We performed differential expression analysis between patients with COVID-19 with or without ARDS with the 3 subsets of monocytes (cMono, intMono, and ncMono) using the FindMarkers function with the parameter “test.use=wilcox”. Significant DEGs were defined with *P*_adj_ < 0.05 calculated by the BH method (64). Gene enrichment analysis of the DEGs was performed using the Metascape online platform (73).

### Data availability.

RNA-Seq, ATAC-Seq, and scRNA-Seq data are accessible in the National Bioscience Database Center (NBDC) Human Database with the accession nos. E-GEAD-551 and E-GEAD-552.

### Statistics.

Statistical analysis was performed by GraphPad Prism 9 (GraphPad Software). The illustrations were created with BioRender.com. Data are represented as the median with interquartile range. Statistical analysis was performed using a repeated-measures 1-way ANOVA with a Greenhouse-Geisser correction and a Bonferroni post hoc test for CyTOF data analysis. The 2-tailed Wilcoxon matched-pairs signed rank test and 2-tailed paired *t* test were used for comparison between 2 groups. Friedman test with Dunn’s post hoc test for multiple comparisons was used for comparisons > 2 groups or time points. *P* < 0.05 was defined as statistically significant.

### Study approval.

All the samples for vaccine cohort and unvaccinated COVID-19 cohort were obtained according to the protocols approved by the local ethics committee of Osaka University Hospital (Suita, Japan) (IRB no. 20118-4) or Osaka University (Suita, Japan) (IRB no. 734-9). Samples were collected after written informed consent was obtained from the participant or his/her representative prior to participation in these studies.

## Author contributions

Y Yamaguchi, YK, and AK contributed to designing research studies. Y Yamaguchi, T Murakami, SA, RE, YS, SN, and Y Yoshimine contributed to conducing experiments. Y Yamaguchi, T Murakami, RE, JNS, T Morita, Y Takeshima, SS, YN, DM, YCL, Y Okita, JF, HH, Y Takeda, JBW, DO, and Y Okada contributed to acquiring data. Y Yamaguchi, YK, JNS, and RE contributed to analyzing data. Y Yamaguchi, YK, JNS, and AK contributed to writing the manuscript.

## Supplementary Material

Supplemental data

## Version 1 10/25/2022

In-Press Preview

## Version 2 11/22/2022

Electronic publication
