# Supplementary material for: Consecutive BNT162b2 mRNA vaccination induces short-term epigenetic memory in innate immune cells
Source: JCI Insight. 2022 Nov 22;7(22):e163347. doi: 10.1172/jci.insight.163347 (PMC9746816; doi:10.1172/jci.insight.163347)
Supplement: Supplemental data [file jciinsight-7-163347-s120.pdf]

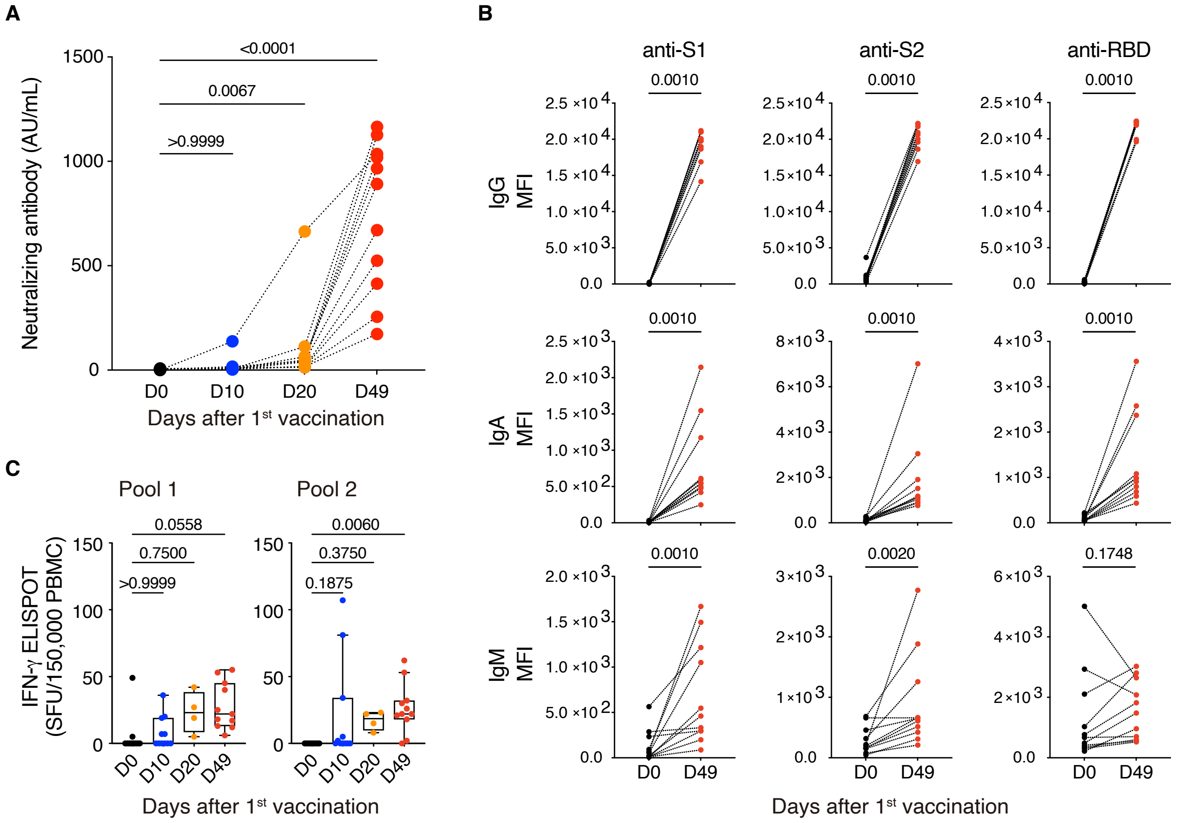

Supplemental Figure 1. The BNT162b2 mRNA vaccine induces humoral and cellular immunity. (A) Titers of neutralizing antibodies against SARS-CoV-2 in serum measured by a chemiluminescence immunoassay before (D0, n = 11) and after (D10, n = 11; D20, n = 5; D49, n = 11) BNT162b2 mRNA vaccination. **(B)** Serum anti-SARS-CoV-2 antigen-specific (spike subunit 1 (S1), spike subunit 2 (S2), and receptor-binding domain (RBD)) IgG, IgA, and IgM, quantified by a bead-based multiplex assay. The data represent the MFI before (D0, n = 11) and after (D49, n = 11) vaccination. **(C)** IFN-γ ELISPOT assay performed with PBMCs collected before (D0, n = 11) and after (D10, n = 11; D20, n = 4; D49, n = 11) vaccination. Pool 1 (left) contained only peptides covering the S1 domain, and Pool 2 (right) contained a mixture of peptides covering some of the S1 domain and peptides covering the RBD and S2 domain. The data represent SFU per 150,000 PBMCs. In all graphs, each dot represents an individual. Black, blue, orange, and red dots represent D0, D10, D20, and D49, respectively (A to C). The box chart indicates the median (middle) and 25th and 75th percentiles (lower and upper), and whiskers indicate the range in all box plots (B). MFI, median fluorescence intensity; ELISPOT, enzyme-linked immunospot; SFU, spot-forming unit. Friedman test followed by Dunn's multiple comparison test was used for all statistical analyses, and each time point (D10, D20, and D49) was compared with the baseline (D0) (A). The two-tailed Wilcoxon matched-pairs signed rank test was used for all statistical analyses (B and C). Bonferroni adjustment was used for multiple comparisons, and each time point (D10, D20, and D49) was compared with the baseline (D0) (C).

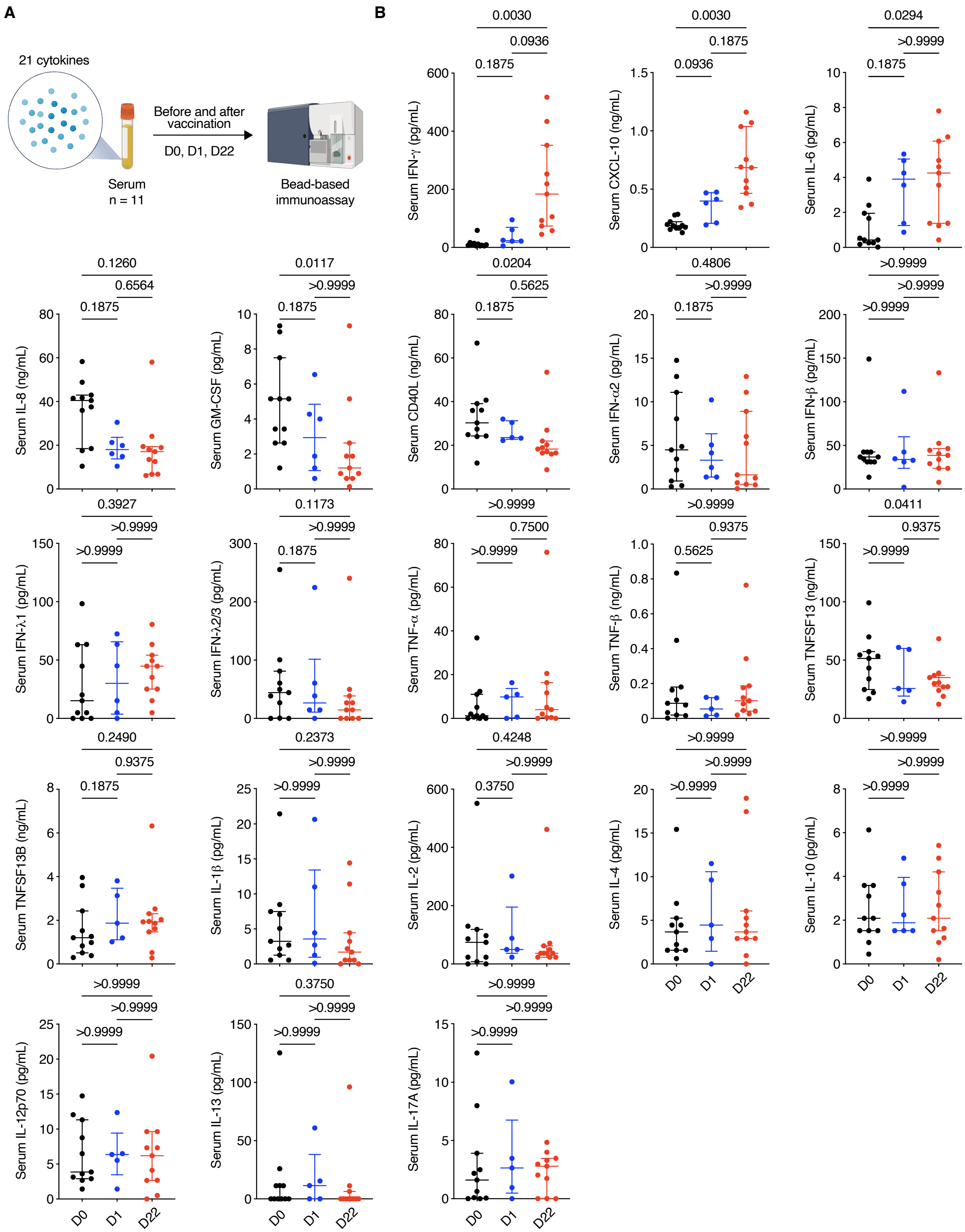

Supplemental Figure 2. Interferon-related cytokines are produced in greater amounts in response to booster vaccination. (A) Overview of the experiment. **(B)** Concentrations of 21

cytokines in serum samples obtained from participants before (D0, n = 11, black) and the day after (D1, n = 6, blue; D22, n = 11, red) BNT162b2 mRNA vaccination, as measured by bead-based immunoassays. In all graphs, each dot represents an individual. Data are shown as the median with the 25th and 75th percentiles. The two-tailed Wilcoxon matched-pairs signed rank test with Bonferroni multiple corrections was used for all statistical analyses, and each time point (D20 and D49) was compared with the baseline.

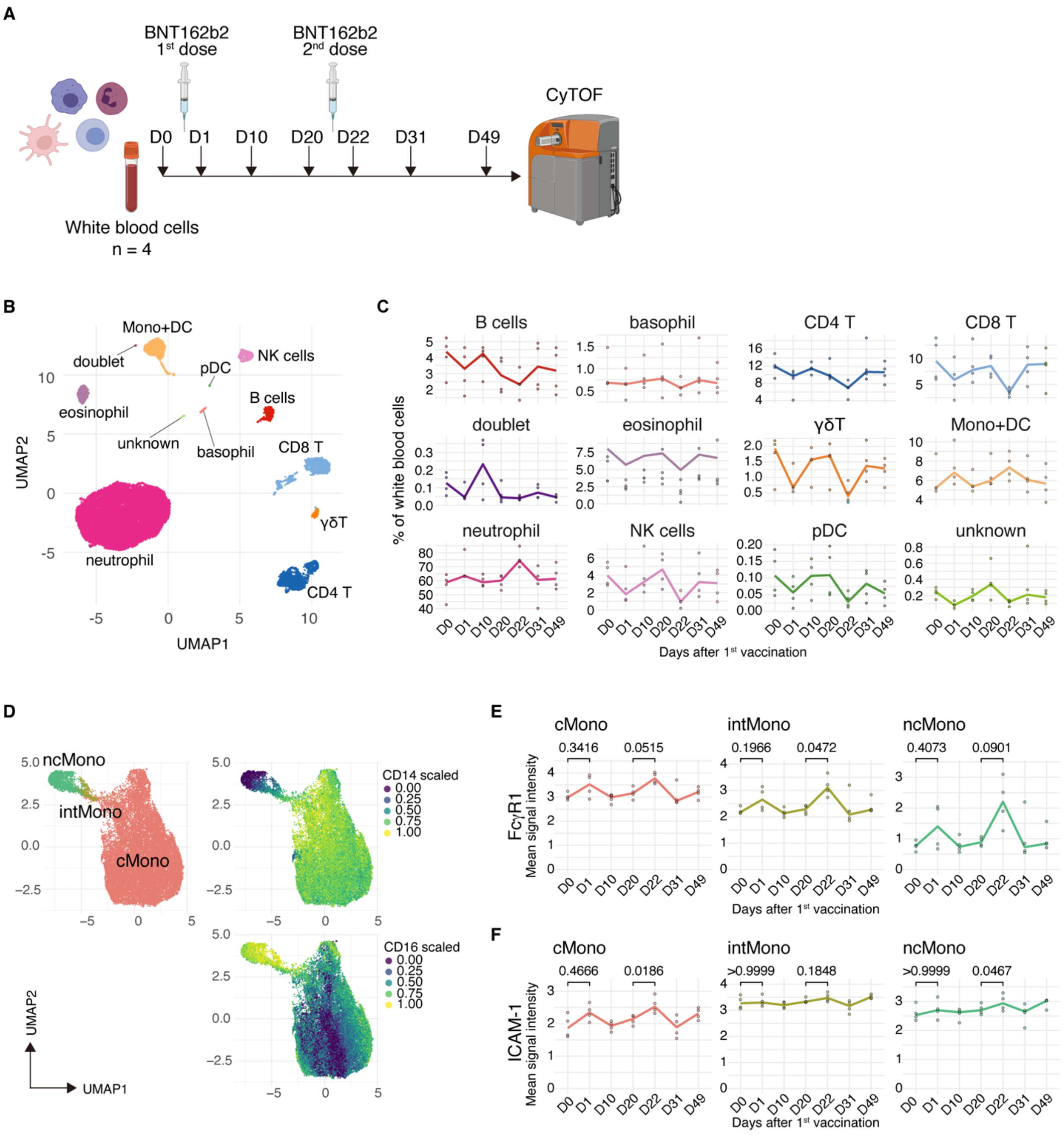

Supplemental Figure 3. CyTOF analysis of monocytes over time. (A) Overview of the CyTOF analysis experiments. Whole blood samples were collected from healthy donors (n = 4) at seven time points and analyzed using CyTOF. **(B)** UMAP showing all identified cell clusters in white blood cells. **(C)** Changes over time in the percentage of each immune cell among white blood cells. **(D)** UMAP clustering of monocytes identified from whole blood samples using CyTOF and UMAP showing the expression levels of CD14 and CD16 in each monocyte cluster. **(E and F)** Changes over time in the expression of FcγR1 (E) and ICAM-1 (F) on classical monocytes (cMono), intermediate monocytes (intMono), and nonclassical monocytes (ncMono), respectively. Statistical analysis was performed using a repeated-measures ANOVA with a Greenhouse-Geisser correction and a Bonferroni post hoc test.

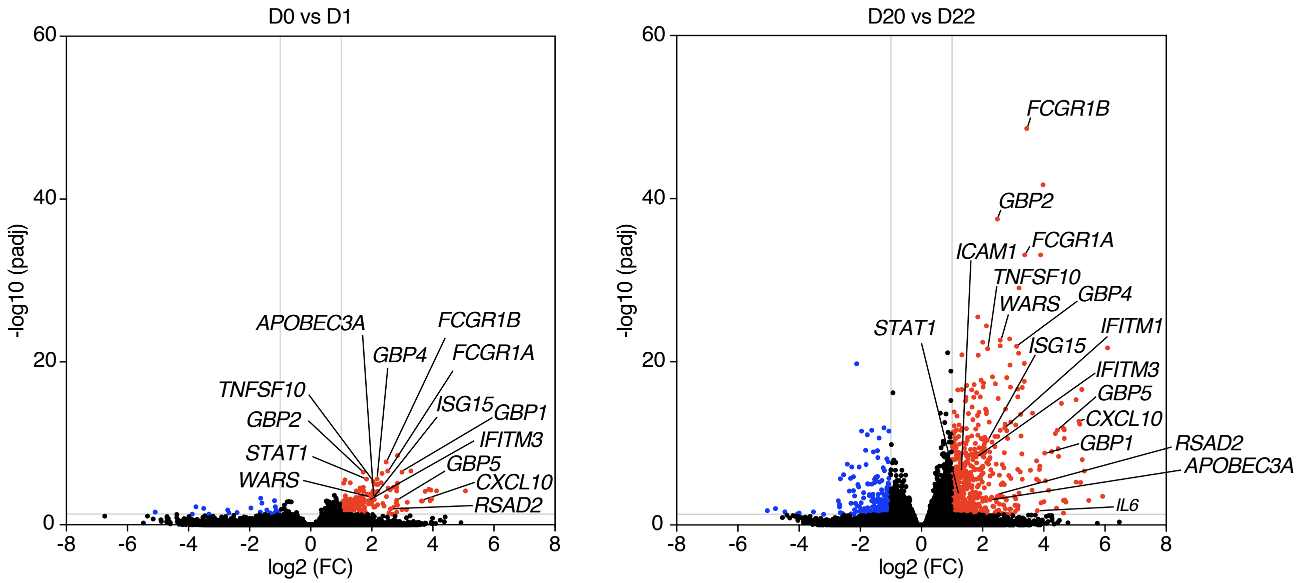

Supplemental Figure 4. The BNT162b2 mRNA vaccine enhanced antiviral and IFN-stimulated gene expression in monocytes. Volcano plot of differentially expressed genes detected by RNA sequencing before and after the first and second vaccination, identified by comparing D1 and D22 to D0 and D20, respectively. Significantly upregulated genes and downregulated genes are indicated by red and blue, respectively (absolute log2-FC > 1 and p.adjust < 0.05). x-axis, log2-fold change; y-axis, log10 adjusted p value.

A

Enrichment analysis of nearest genes in Cluster 2 (Top 100)

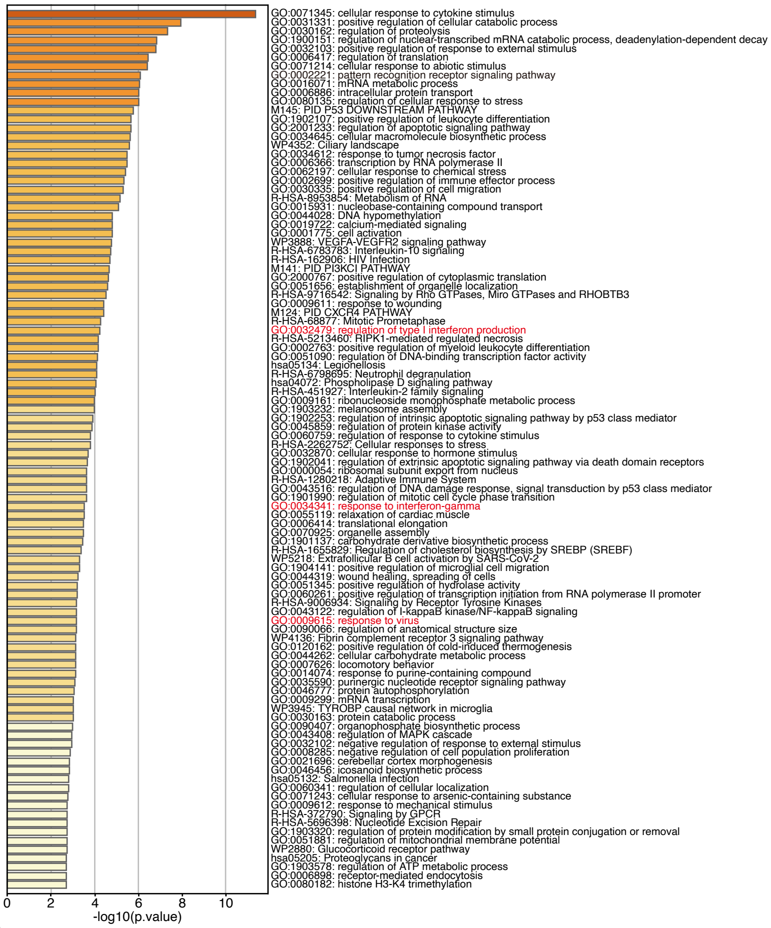

B

MCODE components and corresponding PPI network on Cluster 1 (Top 3 for each component)

MCODE	GO	Description	log10(p.val)
MCODE_1	GO:0062207	regulation of pattern recognition receptor signaling pathway	-5.4
	GO:0051129	negative regulation of cellular component organization	-5.4
	GO:1903320	regulation of protein modification by small protein conjugation or removal	-5.3
MCODE_2	R-HSA-909733	Interferon alpha/beta signaling	-40.0
	R-HSA-913531	Interferon signaling	-33.0
	GO:0051607	defense response to virus	-28.1
MCODE_3	GO:0002831	regulation of response to biotic stimulus	-3.5
	GO:0009615	response to virus	-3.5
	GO:0001819	positive regulation of cytokine production	-3.1
MCODE_4	GO:0019886	antigen processing and presentation of exogenous peptide antigen via MHC class II	-20.4
	GO:0002495	antigen processing and presentation of peptide antigen via MHC class II	-20.2
	GO:0002504	antigen processing and presentation of peptide or polysaccharide antigen via MHC class II	-20.1
MCODE_5	GO:0061013	regulation of mRNA catabolic process	-4.9
	GO:1903311	regulation of mRNA metabolic process	-4.3
	GO:0006417	regulation of translation	-3.7
MCODE_6	hsa04630	JAK-STAT signaling pathway	-9.1
	hsa05145	Toxoplasmosis	-6.7
	R-HSA-1280215	Cytokine Signaling in Immune system	-6.5
MCODE_7	WP49	IL-2 signaling pathway	-8.6
	M122	PID IL2 1PATHWAY	-8.2
	hsa04630	JAK-STAT signaling pathway	-6.8
MCODE_8	R-HSA-9617828	FOXO-mediated transcription of cell cycle genes	-9.8
	R-HSA-9614085	FOXO-mediated transcription	-8.0
	WP710	DNA damage response (only ATM dependent)	-7.3

(related to Figure 2E)

C

Enrichment analysis of nearest genes in Cluster 3

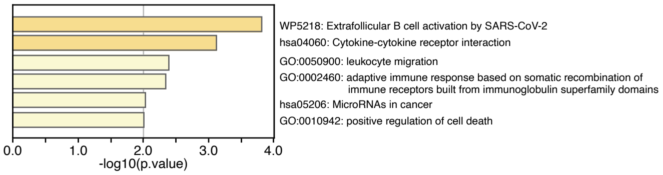

D

Enrichment analysis of nearest genes in Cluster 4

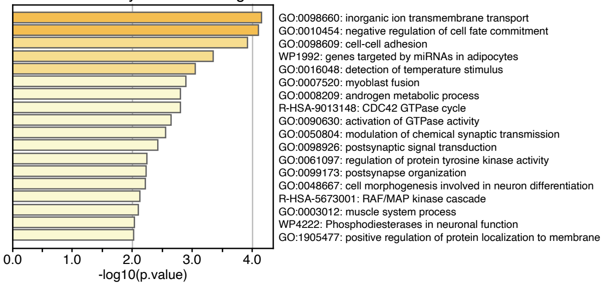

E

ATAC-seq Motifs_closing peaks (D0 vs D49)

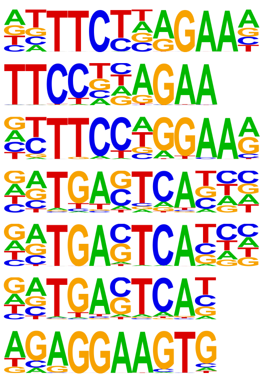

Motif	TF	p-value
	STAT5	1e-36
	STAT6	1e-20
	STAT4	1e-16
	Fosl2	1e-10
	Jun-AP1	1e-9
	JunB	1e-7
	PU.1	1e-3

Supplemental Figure 5. Enrichment analysis of ATAC-seq data. (A) Enrichment analysis of the nearest genes detected in cluster 2 of ATAC-seq data conducted with Metascape (https://metascape.org). The top 100 terms are listed. Innate immune response terms are marked in red. **(B)** MCODE components identified by protein–protein interaction enrichment analysis using Metascape (https://metascape.org) (associated with Fig. 2E). The three best-scoring terms by p value are listed. **(C and D)** Enrichment analysis of the nearest genes detected in clusters 3

(C) and 4 (D) of ATAC-seq data conducted with Metascape (https://metascape.org). **(E)** Known TF binding motifs related to interferon and cytokine production in less accessible regions. The differentially accessible regions were identified in isolated monocytes on D49 compared to D0 (n = 5 per group) using ATAC-seq. TF motifs were detected by hypergeometric optimization of motif enrichment (HOMER) analysis.

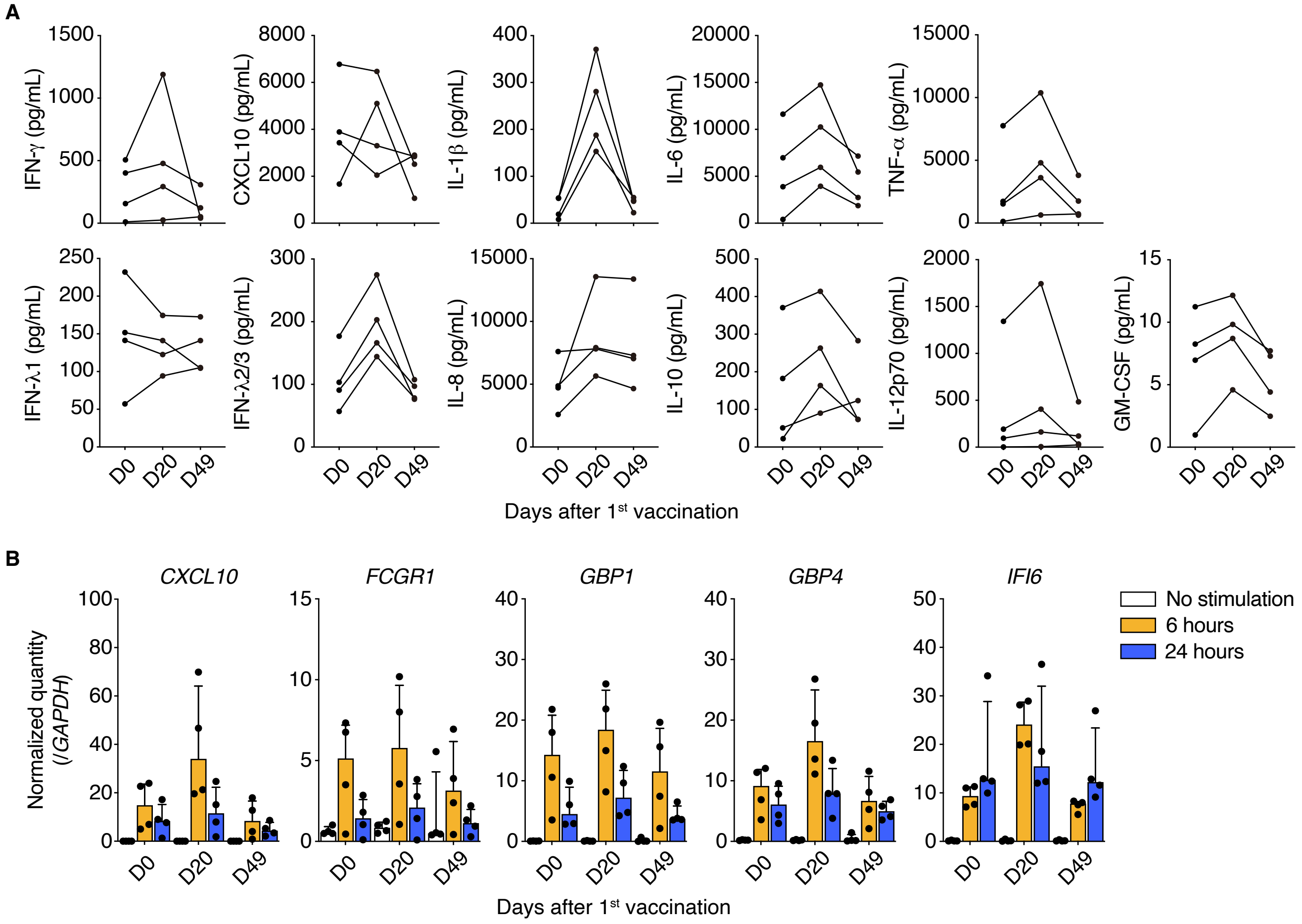

Supplemental Figure 6. Cytokine production and gene expression in monocytes induced by a TLR7/TLR8 agonist. (A) Concentrations of cytokines (IFN-γ, CXCL-10, IL-1β, IL-6, TNF-α, IFN-λ1, IFN-λ2/3, IL-8, IL-10, IL-12p70, and GM-CSF) in the culture supernatant after stimulation of isolated monocytes with R848 for 24 hours (D0, D20, and D49; n = 4 per group), as measured by a bead-based immunoassay. Each dot represents an individual. **(B)** Antiviral and IFN-stimulated gene (*CXCL10*, *FCGR1*, *GBP1*, *GBP4*, and *IFI6*) expression levels were quantified by qPCR before and after stimulation of isolated monocytes with R848 (100 ng/mL) for 6 and 24 hours (left bars, D0; middle bars, D20; right bars, D49; n = 4 per group). The gene expression levels were normalized to those of *GAPDH*. Each dot represents an individual. White, orange, and blue bars show no stimulation, 6 hours and 24 hours after stimulation. ISGs, IFN-stimulated genes.

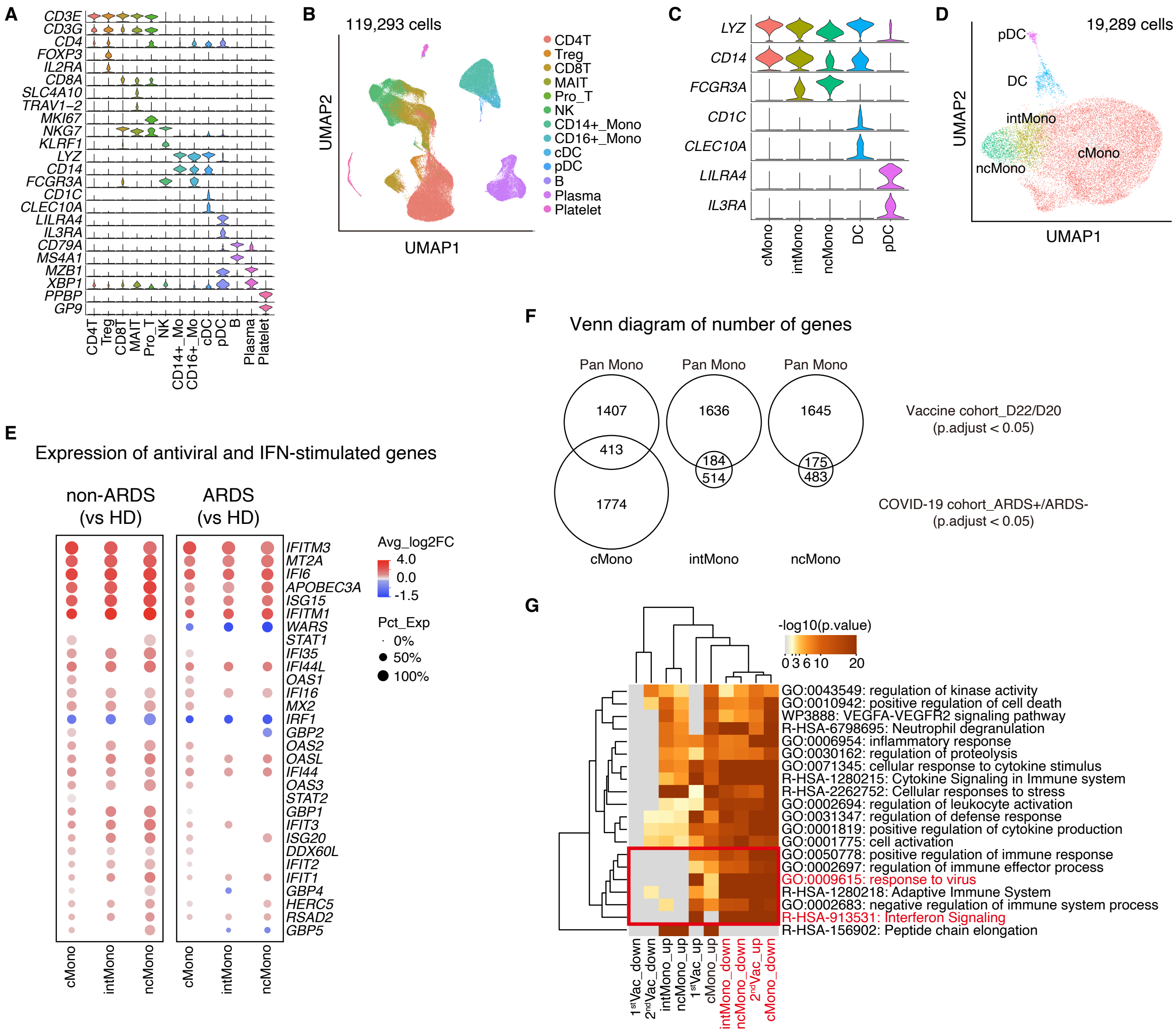

Supplemental Figure 7. scRNA-seq of unvaccinated COVID-19 patients. (A) Violin plots representing the expression of cell markers in 13 clusters. **(B)** UMAP representing all 119,293 cells with manually annotated cell types. **(C)** Violin plots representing the expression of cell markers of innate immune cells (myeloid cells + pDCs) in 5 clusters. **(D)** UMAP clustering of innate immune cells (myeloid cells + pDC) identified by scRNA-seq analysis, colored by manually annotated cell types. **(E)** Dot plots representing significantly differentially expressed antiviral and interferon-stimulated genes in cMono, intMono, and ncMono, comparing non-ARDS COVID-19 patients (left) or COVID-19 patients with ARDS (right) with healthy donors (p.adjust < 0.05). Dot color indicates the average of the log2-FC (Avg_log2FC), and dot size represents the percentage of cells expressing the gene (Pct_Exp). **(F)** Venn diagrams of the number of DEGs in monocytes (cMono, lower left; intMono, lower middle; and ncMono, lower right) identified by scRNA-seq analysis of COVID-19 patients (ARDS versus non-ARDS) and in pan-monocytes (upper) isolated from PBMCs collected before (D20) and after (D22) the second vaccination identified by bulk RNA-seq analysis of vaccinated participants, filtered by p.adjust <

0.05. **(G)** Ontology clusters showing statistically enriched terms using Metascape (https://metascape.org). We selected the term with the best p value within each cluster as its representative term, and they are displayed in a dendrogram. The heatmap is colored by p value for each cluster, and gray cells indicated a lack of enrichment for that term in the corresponding gene list. ARDS, acute respiratory distress syndrome; HD, healthy donor; cMono, classical monocytes; intMono, intermediate monocytes; ncMono, nonclassical monocytes; Pan Mono, pan monocytes (including cMono, intMono, and ncMono); 1^{st}Vac, D0 vs. D1 (vaccine cohort); 2^{nd}VAC, D20 vs. D22 (vaccine cohort); up, upregulated; down, downregulated.

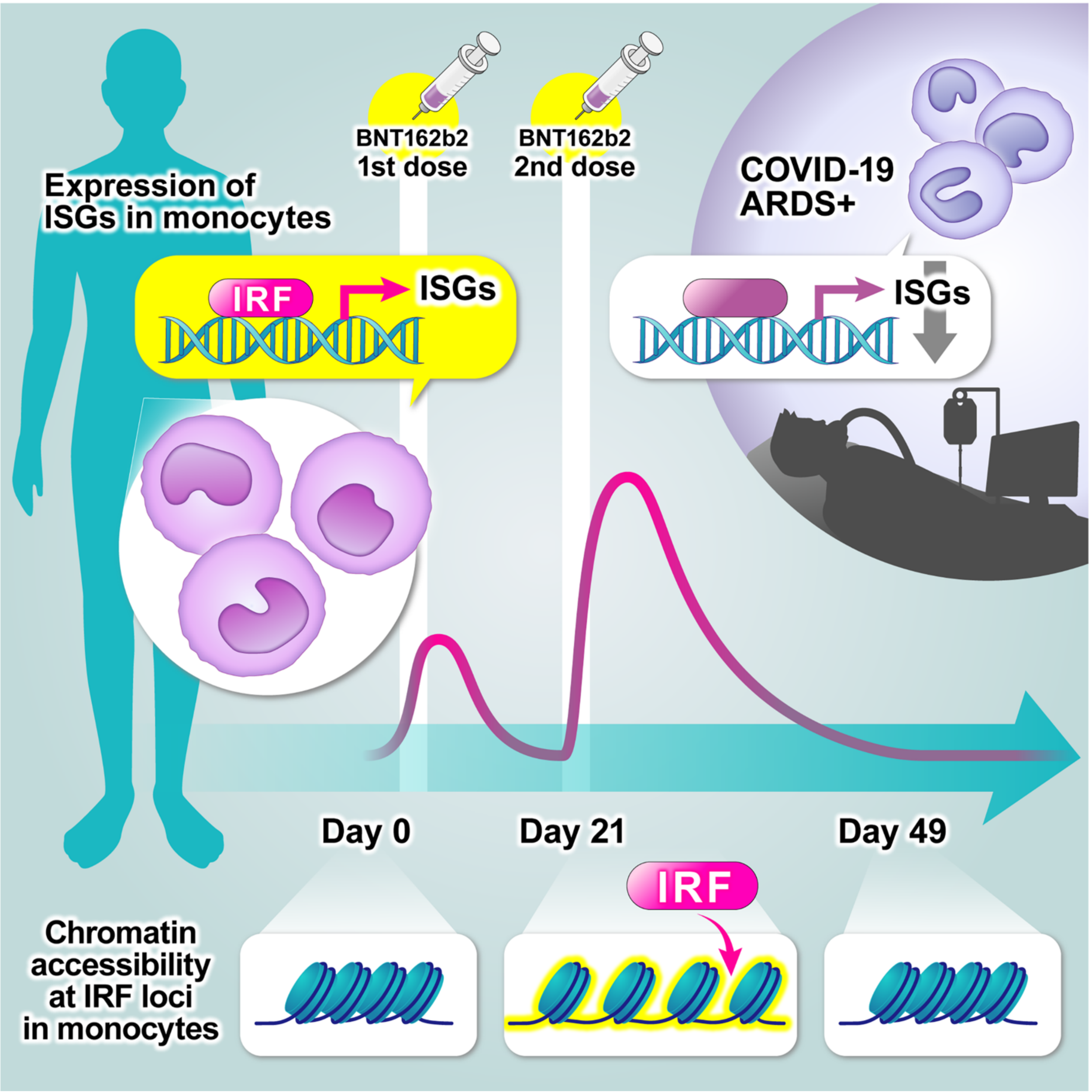

Supplemental Figure 8. Epigenetic short-term memory of innate immune cells after vaccination and COVID-19. The short-term epigenetic reprogramming induced by the BNT162b2 mRNA vaccine leads to a transient enhancement of the type I IFN response in monocytes. Unvaccinated COVID-19 patients, especially those with ARDS, had impaired the expression of ISGs, which were strongly correlated with the genes upregulated vaccination. ARDS, acute respiratory distress syndrome; IRF, interferon-regulatory factor; ISG, IFN-stimulated gene.

Supplemental Table 1. Healthy donor characteristics in the vaccine study.

Sample	Donor	Sex	Age (years)	BMI	SARS-CoV-2 Infection	CyTOF (Fig. 1)	RNA-seq (Fig. 1)	ATAC-seq (Fig. 2)	Restimulation (Fig. 3)
Serum/PBMC	HD_01	Male	32	22.1	No	No	No	No	No
Serum/PBMC	HD_02	Male	31	20	No	No	No	No	No
Serum/PBMC/WBC	HD_03	Male	36	22.2	No	Yes	Yes	Yes	Yes
Serum/PBMC	HD_04	Male	35	22.7	No	No	No	No	No
Serum/PBMC/WBC	HD_05	Male	42	22.5	No	Yes	Yes	Yes	No
Serum/PBMC	HD_06	Male	33	20.1	No	No	No	No	No
Serum/PBMC	HD_07	Male	38	24.7	No	No	No	No	No
Serum/PBMC	HD_08	Male	34	24.1	No	No	No	Yes	Yes
Serum/PBMC	HD_09	Male	40	23.9	No	No	No	No	No
Serum/PBMC/WBC	HD_10	Male	37	23.1	No	Yes	Yes	Yes	Yes
Serum/PBMC/WBC	HD_11	Male	42	24.5	No	Yes	Yes	Yes	Yes

Supplemental Table 2. Donor characteristics in the unvaccinated COVID-19 patient study for scRNA-seq analysis. COPD, chronic obstructive pulmonary disease; CO, coronavirus disease 2019 (COVID-19); DCM, dilated cardiomyopathy; DL, dyslipidemia; DM, type 2 diabetes mellitus; HC, healthy control; HT, hypertension; IP, Interstitial pneumonia; LAM, lymphangioleiomyomatosis.

ID	HC_01	HC_02	HC_03	HC_04	HC_05	CO_01	CO_02	CO_03	CO_04	CO_05	CO_06	CO_07	CO_08	CO_09	CO_10	CO_11	CO_12	CO_13	CO_14	CO_15	CO_16
**Status**	Healthy	Healthy	Healthy	Healthy	Healthy	COVID-19	COVID-19	COVID-19	COVID-19	COVID-19	COVID-19	COVID-19	COVID-19	COVID-19	COVID-19	COVID-19	COVID-19	COVID-19	COVID-19	COVID-19	COVID-19
**ARDS**	-	-	-	-	-	No	No	Yes	No	Yes	Yes	No	No	No	Yes	Yes	Yes	Yes	No	Yes	No
**Severity (WHO classification)**	-	-	-	-	-	Severe	Severe	Critical	Severe	Critical	Critical	Severe	Severe	Severe	Critical	Critical	Critical	Critical	Severe	Critical	Severe
**Age (years)**	55	62	60	64	58	50	64	82	62	84	73	39	78	40	80	79	56	80	73	64	58
**Sex**	Female	Female	Male	Male	Male	Male	Male	Male	Male	Female	Male	Female	Male	Male	Female	Male	Male	Male	Male	Female	Female
**Body mass index (BMI)**	NA	NA	NA	NA	NA	24.5	24.2	23.4	26	29	23.5	15.6	24.2	34.7	24.1	23.1	23.3	23.2	20	22.1	49.9
**Smoking (Brinkman index)**	NA	NA	NA	NA	NA	800	460	800	1080	0	NA	18	0	NA	NA	3000	NA	NA	560	NA	0
**Underlying disease**	No	No	No	No	No	DM, HU	DCM	IP, HT,	No	HT, DM	No	LAM	HT	No	No	COPD	HT, DL	HT, DL	HT	No	Carcinoma
**Clinical outcome**	-	-	-	-	-	Recovered	Recovered	Deceased	Recovered	Recovered	Deceased	Recovered	Recovered	Recovered	Deceased	Recovered	Recovered	Recovered	Recovered	Deceased	Recovered
**Blood test**																					
**White blood cell (x10^9/L)**	NA	NA	NA	NA	NA	8.55	2.84	14.78	4.84	11.66	12.88	2.15	6.35	3.68	14.23	6.62	3.06	7.01	5.81	2.92	0.82
**Haemoglobin (g/L)**	NA	NA	NA	NA	NA	131	114	114	129	10.3	133	144	145	142	137	136	145	130	128	121	112
**Platelet (x10^9/L)**	NA	NA	NA	NA	NA	315	86	175	296	255	155	138	255	284	217	144	371	192	217	143	144
**Neutrophil (%)**	NA	NA	NA	NA	NA	75.1	83.8	89.2	88.9	95.60	87.8	65.6	77.5	66.4	94.1	91	91.8	89	65.1	89.1	56.1
**Lymphocyte (%)**	NA	NA	NA	NA	NA	17.9	13	5.9	7.6	3	6.5	23.7	16.9	29.3	3.6	6.3	5.9	7.1	18.8	8.2	37.8
**Monocyte (%)**	NA	NA	NA	NA	NA	6.9	3.2	4.8	3.5	1.3	5.5	9.8	5.4	3.5	2.2	2	2.3	3.9	15.7	2.4	4.9
**Eosinophil (%)**	NA	NA	NA	NA	NA	0.0	0.0	0.0	0.0	0.0	0.0	0.0	0.0	0.3	0.0	0.2	0.0	0	0.2	0.0	0.0
**Basophil (%)**	NA	NA	NA	NA	NA	0.1	0	0	0.0	0.1	0	0.9	0.2	0.5	0.1	0.5	0.0	0	0.2	0.3	1.2
**D-dimer (μg/mL)**	NA	NA	NA	NA	NA	2.94	3.01	0.75	1.35	0.64	0.67	0.82	4.49	1.33	1.58	3.76	4.53	0.98	0.59	0.81	0.97
**LDH (IU/L)**	NA	NA	NA	NA	NA	335	395	423	360	289	551	386	318	345	368	644	392	439	305	400	238
**C-reative protein (mg/L)**	NA	NA	NA	NA	NA	17.3	21.1	9.2	17.6	6.52	38.8	6.5	41.4	28.4	36.3	80.3	103	48	45.7	157.4	34.3
**IgG (g/L)**	NA	NA	NA	NA	NA	11.09	17.48	11.07	13.78	10.24	8.74	10.87	9.87	13.32	10.59	9.42	9.76	13.54	9.58	13.54	17.97
**IgA (g/L)**	NA	NA	NA	NA	NA	3.65	2.58	1.46	0.57	1.99	2.96	1.73	2.34	2.35	3.18	2.02	2.14	1.41	3.44	3.93	3.91
**IgM (g/L)**	NA	NA	NA	NA	NA	1.07	0.55	0.42	0.31	1.07	1.03	1.16	1.16	1.37	0.96	0.31	0.79	0.66	0.73	0.74	0.83
**Ferritin (ng/mL)**	NA	NA	NA	NA	NA	2820	955	387	1234	151	534	42	NA	NA	443	507	771	655	1287	348	847
**PCR test**																					
**Blood**	-	-	-	-	-	NA	Positive	Positive	Positive	Positive	Positive	Negative	Positive	Positive	Positive	Positive	Negative	Positive	Negative	Positive	Negative
**Sputum**	-	-	-	-	-	NA	Positive	Positive	Positive	Positive	Positive	NA	Positive	Positive	NA	NA	Positive	Positive	NA	NA	NA
**Nasopharyngeal swab**	-	-	-	-	-	Positive	NA	Positive	Positive	Positive	Positive	Positive	Positive	Positive	Positive	Positive	Positive	Positive	Positive	Positive	Positive

Supplemental Table 3. Information about the antibody panel used for CyTOF analysis.

Isotope/Metal	Target	Clone	Vender	Cat#	Conjugation	Concentration
89Y	CD45	HI30	Fluidigm	201325	commercial	1:100
103Rh	Cell-ID intercalator		Fluidigm	201325	commercial	1:100
106Cd	IFN-$\alpha\beta$_R2	493715	R&D	MAB4015	in-house	1:100
110Cd	IFN-$\alpha\beta$_R1	85228	R&D	MAB245	in-house	1:100
111Cd	NRP-1	446921	R&D	MAB3870	in-house	1:100
112Cd	ICAM-1	BBIG-I1	R&D	BBA3	in-house	1:100
114Cd	FCGR1	10.1	R&D	MAB1257	in-house	1:100
116Cd	CXCR1	42705	R&D	MAB330	in-house	1:100
141Pr	CCR6	G034E3	Fluidigm	201325	commercial	1:100
142Nd	FCAR	488032	R&D	MAB3939	in-house	1:100
143Nd	CD123	6H6	Fluidigm	201325	commercial	1:100
144Nd	CD19	HIB19	Fluidigm	201325	commercial	1:100
145Nd	CD4	RPA-T4	Fluidigm	201325	commercial	1:100
146Nd	CD8a	RPA-T8	Fluidigm	201325	commercial	1:100
147Sm	CD11c	Bu15	Fluidigm	201325	commercial	1:100
148Nd	CD16	3G8	Fluidigm	201325	commercial	1:100
149Sm	CD45RO	UCHL1	Fluidigm	201325	commercial	1:100
150Nd	CD45RA	HI100	Fluidigm	201325	commercial	1:100
151Eu	CD161	HP-3G10	Fluidigm	201325	commercial	1:100
152Sm	CCR4	L291H4	Fluidigm	201325	commercial	1:100
153Eu	CD25	BC96	Fluidigm	201325	commercial	1:100
154Sm	CD27	O323	Fluidigm	201325	commercial	1:100
155Gd	CD57	HCD57	Fluidigm	201325	commercial	1:100
156Gd	CXCR3	G025H7	Fluidigm	201325	commercial	1:100
158Gd	CXCR5	J252D4	Fluidigm	201325	commercial	1:100
159Tb	CD147	TRA-1-85	R&D	MAB3195	in-house	1:100
160Gd	CD28	CD28.2	Fluidigm	201325	commercial	1:100
161Dy	CD38	HB-7	Fluidigm	201325	commercial	1:100
162Dy	CD44	IM7	Fluidigm	3162030B	commercial	1:100
163Dy	CD56	NCAM16.2	Fluidigm	201325	commercial	1:100
164Dy	TCRγδ	B1	Fluidigm	201325	commercial	1:100
165Ho	Sema4D	758726	R&D	MAB74701	in-house	1:100
166Er	CD294	BM16	Fluidigm	201325	commercial	1:100
167Er	CCR7	G043H7	Fluidigm	201325	commercial	1:100
168Er	CD14	63D3	Fluidigm	201325	commercial	1:100
169Tm	Sema6D	257510	R&D	MAB2095	in-house	1:100
170Er	CD3	UCHT1	Fluidigm	201325	commercial	1:100
171Yb	CD20	2H7	Fluidigm	201325	commercial	1:100
172Yb	CD66b	G10f5	Fluidigm	201325	commercial	1:100
173Yb	HLA-DR	LN3	Fluidigm	201325	commercial	1:100
174Yb	IgD	IA6-2	Fluidigm	201325	commercial	1:100
175Lu	CXCR2	48311	R&D	MAB331	in-house	1:100
176Yb	CD127	A019D5	Fluidigm	201325	commercial	1:100
209Bi	PD-L1	MIH1	Fluidigm	3209014B	commercial	1:100

Supplemental Table 4. Primer sequences for quantitative PCR.

Gene	Forward (5'-3')	Reverse (5'-3')	NCBI Reference Sequence
APOBEC3A	GACAATGGCACCTCGGTCAAGA	CCAACTGCAAAGAAGGAACCAGG	NM_145699
CXCL10	TTCCTGCAAGCCAATTTTGT	TTCTTGATGGCCTTCGATTC	NM_001565
FCGR1	ATACAGGTGCCAGAGAGGTCTC	CCAGCTTATCCTTCCACGCATG	NM_000566
GAPDH	GTCTCCTCTGACTTCAACAGCG	ACCACCCTGTTGCTGTAGCCAA	NM_002046
GBP1	TATTGCCCACTATGAACAGCAGAT	TAGCTGGGCCGCTAACTCC	NM_002053.3
GBP4	TAAGCGGCTTTCAGAGCACC	GACCTCGTTTGCCTTAACTCC	NM_052941.5
GBP5	CTGTCTGCCATTACGCAACCTG	GTGTGAGACTGCACCGTAGATG	NM_001134486
IFI6	TGATGAGCTGGTCTGCGATCCT	GTAGCCCATCAGGGCACCAATA	NM_022873
IFITM1	GGCTTCATAGCATTCGCCTACTC	AGATGTTCAGGCACTTGGCGGT	NM_003641
ISG15	CGCAGATCACCCAGAAGATCG	TTCGTCGCATTTGTCCACCA	NM_005101.4
TNFSF10	TGGCAACTCCGTCAGCTCGTTA	AGCTGCTACTCTCTGAGGACCT	NM_003810
WARS	GGACATCATCGCCTGTGGCTTT	AGTCGCTGTCAGTGAAGCCGAA	NM_004990
